# The Evolutionary Dynamics of Repetitive DNA and Its Impact on the Genome Diversification in the Genus *Sorghum*

**DOI:** 10.3389/fpls.2021.729734

**Published:** 2021-08-12

**Authors:** Yi-Tzu Kuo, Takayoshi Ishii, Jörg Fuchs, Wei-Hsun Hsieh, Andreas Houben, Yann-Rong Lin

**Affiliations:** ^1^Leibniz Institute of Plant Genetics and Crop Plant Research (IPK), Gatersleben, Germany; ^2^Department of Agronomy, National Taiwan University, Taipei, Taiwan; ^3^Arid Land Research Center, Tottori University, Tottori, Japan; ^4^World Vegetable Center, Tainan, Taiwan

**Keywords:** satellite DNA, centromere, genome evolution, CENH3, long terminal repeat (LTR), dryland crop, *Sorghum*

## Abstract

Polyploidization is an evolutionary event leading to structural changes of the genome(s), particularly allopolyploidization, which combines different genomes of distinct species. The tetraploid species, *Sorghum halepense*, is assumed an allopolyploid species formed by hybridization between diploid *S. bicolor* and *S. propinquum*. The repeat profiles of *S. bicolor*, *S. halepense*, and their relatives were compared to elucidate the repeats’ role in shaping their genomes. The repeat frequencies and profiles of the three diploid accessions (*S. bicolor*, *S. bicolor* ssp. *verticilliflorum*, and *S. bicolor* var. *technicum*) and two tetraploid accessions (*S. halepense*) are similar. However, the polymorphic distribution of the subtelomeric satellites preferentially enriched in the tetraploid *S. halepense* indicates drastic genome rearrangements after the allopolyploidization event. Verified by CENH3 chromatin immunoprecipitation (ChIP)-sequencing and fluorescence *in situ* hybridization (FISH) analysis the centromeres of *S. bicolor* are mainly composed of the abundant satellite SorSat137 (CEN38) and diverse CRMs, Athila of Ty3_gypsy and Ty1_copia-SIRE long terminal repeat (LTR) retroelements. A similar centromere composition was found in *S. halepense*. The potential contribution of *S. bicolor* in the formation of tetraploid *S. halepense* is discussed.

## Introduction

Large genomes are rich in various repetitive DNAs; for example, up to 85 and 90% of the maize and wheat genomes are composed of repeats (Li et al., 2004; Schnable et al., 2009). Although once thought to be “junk DNA,” repetitive DNA has been later found to be involved in regulating gene expression, maintenance of chromosomal integrity, and genome stability (Bennetzen and Wang, 2014; Mehrotra and Goyal, 2014; Garrido-Ramos, 2015). Compared to coding sequences, repetitive DNAs are considered as fast-evolving genome components. Their variable abundance, high sequence variations and distinct chromosomal distributions contribute to genome divergence among species.

Repetitive DNAs can be classified into two major types, tandem repeats (also referred to as satellite DNA) and transposable elements (TEs), according to their structural organization and sequence composition (Kubis et al., 1998). Tandem repeats, which repeat unit arrays in a head-to-tail manner, preferentially cluster at specific chromosome regions, such as (peri)centromeres, (sub)telomeres, and distinct intercalary regions. TEs tend to intersperse with other sequences and scatter throughout entire genome but can also accumulate at specific chromosomal regions, as, e.g. (peri)centromeres. TEs are further divided into DNA transposons and retrotransposons, which transpose within the genome *via* either cut-and-paste or copy-and-paste mechanisms, respectively. Retrotransposons are the most abundant TEs in eukaryotes. Of them, the long terminal repeat (LTR) retrotransposons, subclassified into Ty1_copia and Ty3_gypsy, were reported to be present throughout the entire plant kingdom (Kumar and Bennetzen, 1999).

Centromeric and pericentromeric regions are the hotspots for repeat accumulation. In species like rice (Cheng et al., 2002), maize (Zhong et al., 2002), and barley (Houben et al., 2007), both satellite repeats and retrotransposons are enriched in these areas and interact partly with the centromere-defining centromeric histone H3 variant CENH3. The centromeric satellite is often the most abundant tandem repeat in a genome, and its corresponding monomer unit is highly variable in sequence composition and length between species (Melters et al., 2013). The most common monomer sizes of centromeric satellites are 140 ∼ 180 and 300 ∼ 360 bp, representing mono- and dinucleosomes. However, centromeric satellite units with a length of only 20 bp as in *Astragalus sinicus* (Tek et al., 2011) and longer units up to 2,979 bp in *Pisum fulvum* (Robledillo et al., 2020) were also found.

Transposable elements not only occupy a significant portion of eukaryotic genomes but also play a role in centromere evolution (Hartley and O’Neill, 2019). The insertion of centromeric retroelements is related to the birth of new satellite families in *Arabidopsis thaliana* (Kapitonov and Jurka, 1999) and *Aegilops speltoides* (Cheng and Murata, 2003). In *A. thaliana*, they are also involved in the transcriptional regulation of centromeric satellite DNA (May et al., 2005). The interaction between satellite repeats and retrotransposons drives the rapid sequence changes in plant centromeres, especially after interspecific hybridization or polyploidization (Yang et al., 2018; Su et al., 2019).

The genus *Sorghum* belongs to the Poaceae family and is divided into five subgenera: *Eusorghum*, *Parasorghum*, *Heterosorghum*, *Chaetosorghum*, and *Stiposorghum* (Garber, 1950). In the subgenus *Eusorghum*, the cultivated species, *S. bicolor* (L.) Moench (2*n* = 2x = 20) originated from Africa and is known for its drought tolerance and broad adaptation. As one of the top five cereal crops, it is used for multiple purposes, like staple food, forage crop, and biofuel. Another cultivated species, *S. bicolor* var. *technicum* (Körn.) Stapf ex Holland, is used for making brooms. In Taiwan, the commonly discovered wild relatives of the cultivated sorghums are *S. bicolor* ssp. *verticilliflorum* (Steud.) de Wet ex Wiersema & J. Dahlb (2*n* = 2x = 20) and *S. halepense* (L.) Pers. (also called Johnsongrass, 2*n* = 40) (De Wet, 1978), and occasionally *S. propinquum* (Kunth) Hitchc as well. The tetraploid *S. halepense* is likely an allopolyploid species formed by hybridization between *S. bicolor* and *S. propinquum* based on the meiotic studies of *S. bicolor* × *S. halepense* hybrids (Tang and Liang, 1988), genomic sequences (Paterson et al., 2020), and it rhizomatous nature (Paterson et al., 1995). Phylogenetically *S. halepense* is closer to *S. bicolor* (Price et al., 2005), while the *S. propinquum*-derived rhizomes make *S. halepense* a noxious weed with an almost worldwide distribution.

The first *S. bicolor* genome was assembled by Paterson et al. (2009) and has been refined by re-sequencing and optical mapping (Deschamps et al., 2018; McCormick et al., 2018). Genome assemblies demonstrated that the heterochromatic, pericentromeric regions of *S. bicolor* are enriched in repetitive elements (Kim et al., 2005; Paterson et al., 2009; McCormick et al., 2018). The satellite repeat CEN38 (Miller et al., 1998b; Zwick et al., 2000) and the retrotransposon-related DNA element Sau3A9 (Miller et al., 1998a) were found to be associated with the centromeres. Nevertheless, variation in the repeat composition and distribution among different *Sorghum* species remain largely unknown.

In this study, we *in silico* identified high-copy repeats and determined their chromosomal distribution to resolve the relationship and genome diversification of diploid *S. bicolor* and allotetraploid *S. halepense* accessions. The observed polymorphic distribution of the subtelomeric satellites being more abundant in the tetraploids indicates drastic genome rearrangements after the allopolyploidization event forming *S. halepense*. Application of a *Sorghum*-specific CENH3 antibody in combination with chromatin immunoprecipitation (ChIP) sequencing and fluorescence *in situ* hybridization (FISH) resulted in the identification of a centromere-specific satellite and evolutionarily conserved centromere-associated TEs. The impact of dynamic repetitive DNAs in the genome of the five related *Sorghum* genomes is discussed.

## Materials and Methods

### Plant Materials

The diploid species *Sorghum bicolor* accession “V9,” *S. bicolor* ssp. *verticilliflorum* accession “WL” and *S. bicolor* var. *technicum* accession “YL” and two tetraploid accessions of *S. halepense* (accession “TT” and “US”) were grown under greenhouse condition in the Department of Agronomy, National Taiwan University, Taiwan. The collection sites or source of seeds are described in Table 1.

**TABLE 1:** Information of the *Sorghum* materials and the genome size.

**Sorghum sample**	**Abbreviation**	**Chromosome number**	**Genome size**		**Collection site or source of seeds**
			**pg/2C**	**Mb/1C**	
*Sorghum bicolor* “V9”	SbV9	2*n* = 2x = 20	1.61	789	National Taiwan University, Taiwan
*Sorghum bicolor* ssp. *verticilliflorum* “WL”	SbWL	2*n* = 2x = 20	1.64	800	Wanluan, Pingtung, Taiwan
*Sorghum bicolor* var. *technicum* “YL”	SbYL	2*n* = 2x = 20	1.63	795	Yuli, Hualien, Taiwan
*Sorghum halepense* “TT”	ShTT	2*n* = 4x = 40	3.16	1,547	Taitung City, Taitung, Taiwan
*Sorghum halepense* “US”	ShUS	2*n* = 4x = 40	3.10	1,512	Texas, United States

### Flow Cytometry

For nuclei isolation, approximately 0.5 cm^2^ of fresh leaf tissue was chopped together with equivalent amounts of leaf tissue of the internal reference standard, *Glycine max* (L.) Merr. convar. max var. max, Sorte Cina 5202 (Gatersleben GeneBank accession number: SOJA 392; 2.21 pg/2C), in a petri dish using the reagent kit “CyStain PI Absolute P” (Sysmex-Partec) following the manufacturer’s instructions. The resulting nuclei suspension was filtered through a 50-μm CellTrics filter (Sysmex-Partec) and measured on a BD Influx cell sorter (BD Biosciences). Six independent measurements were performed for each genotype. The absolute DNA content (pg/2C) was calculated based on the values of the G1 peak means and the corresponding genome size (Mbp/1C), according to Dolezel et al. (2003).

### DNA Extraction and Genome Sequencing

The genomic DNA of *Sorghum* plants was extracted from the young leaves using DNeasy Plant Mini Kit (QIAGEN). Low-pass genome sequencing was performed, generating 2 × 150 bp paired-end (PE) reads using NovaSeq 6000 system (Illumina) by Novogene (China). At least 4 GB raw PE reads were generated for each *Sorghum* sample.

### Analysis of Repetitive Genome Fractions Using RepeatExplorer and TAREAN

The quality of the PE reads was assessed by FastQC (Andrews, 2010) implanted in the RepeatExplorer pipeline^1^ and filtered by quality with 95% of bases equal to or above the cut-off value of 10, followed by an overlap check. Non-overlapped PE reads equivalent to 0.3× genome coverage were sampled and employed to identify, characterize, and quantify the repetitive elements in each individual genome by the graph-based clustering method using RepeatExplorer (Novák et al., 2010, 2013). Clustering was performed by default setting with 90% of similarity over 55% of the read length. The comparative clustering analysis was performed based on 0.1× genome coverage of qualified PE reads from each *Sorghum* sample according to the protocol in Novák et al. (2020). The automatic annotation of repeat clusters was inspected manually and revised if necessary, and the genome proportion of each repeat type was recalculated. The monomer sequence of putative satellites and LTR elements were reconstructed by TAREAN (TAndem REpeat ANalyzer) (Novák et al., 2017).

### Phylogenetic Analysis

The CENH3 protein sequences of *S. bicolor* and other plant species were downloaded from NCBI GenBank (Supplementary Table 1). They were first aligned using ClustalW implanted in MEGA X by default setting (Thompson et al., 1994; Kumar et al., 2018), and the phylogenetic relationship was inferred by the maximum likelihood method on the IQ-Tree web server^2^ (Trifinopoulos et al., 2016). The tree was visualized and exported using Interactive Tree Of Life (iTOL^3^) (Letunic and Bork, 2007, 2019).

### Probe DNA Preparation

The primers used to amplify probe DNA were designed using Primer3 based on the sequences of satellite monomer or LTR integrase domain, identified in NCBI CD-Search^4^ (Marchler-Bauer and Bryant, 2004; Lu et al., 2020). The sequence of primers and repeat clusters are listed in Supplementary Tables 2, 3. The probe DNAs were amplified in a mixture of 50 ng genomic DNA, 1× PCR buffer, 0.25 mM of each dNTP, 0.4 mM of each primer, 1.5 U Taq polymerase (QIAGEN), in a total of 50 μl with a program of 95°C for 5 min, 35 cycles of 95°C for 30 s, 55°C for 1 min, and 72°C for 1 min, followed by 72°C for 5 min. The purified probe DNAs were then labeled with ATTO488-dUTP or ATTO550-dUTP using the Fluorescent Nick Translation Labeling kits (Jena Bioscience).

### Fluorescence *in situ* Hybridization

Root tips were pretreated with 2 mM 8-hydroxyquinoline at room temperature (RT) for 4–5 h and fixed in freshly prepared 3:1 (v/v) ethanol: glacial acetic acid at RT, overnight. Mitotic chromosome spreads were prepared as described in Aliyeva-Schnorr et al. (2015). Slides were first treated with 45% acetic acid at RT for 10 min, followed by 0.1% pepsin/0.01 N HCl at 37°C for 10 min and post-fixed in 4% paraformaldehyde (PFA) at RT for 10 min. The hybridization mixture contained 50% (v/v) formamide, 10% (w/v) dextran sulfate, 2× SSC, and 5 ng/μl of each probe. Chromosomal DNA and probes were denatured at 75°C for 2 min, and hybridization was performed at 37°C for 15–24 h. The final stringent wash was in 2× SSC at 57°C for 20 min, followed by dehydration in 70, 90, and 100% ethanol series for 3 min each. Chromosomes were counterstained by 10 μg/ml 4′,6-diamidino-2-phenylindole (DAPI) in Vectashield Antifade Mounting Medium (Vector Laboratories).

### Indirect Immunostaining

Polyclonal antibodies against the CENH3 protein of *S. bicolor* were produced by using the N-ERAGGASTSATPERRNAGT-C peptide. The peptide synthesis, immunization of rabbits, and peptide affinity purification of antisera were performed by LifeTein.^5^ For slide preparation, root tips were fixed in 4% PFA in 1× phosphate-buffered saline (PBS) under vacuum at 4°C for 10 min, followed by 20 min at 4°C without vacuum. Roots were washed with ice-cold 1× PBS for 3 min twice, and digested with an enzyme cocktail composed of 1% (w/v) pectolyase (Sigma), 0.7% (w/v) cellulase “ONOZUKA” R-10 (Yakult), 0.7% cellulase (CalBioChem), and 1% cytohelicase (Sigma) in 1× PBS for 60 min at 37°C in a humid chamber. Roots were subsequently washed in ice-cold 1× PBS for 3 min twice. Root tips were then squashed in 1× PBS between slide and coverslip. After freezing in liquid nitrogen, coverslips were removed, and slides were kept in ice-cold 1× PBS. For immunostaining, the SbCENH3 antibody (diluted 1:2,000) was applied at 4°C overnight. Slides were washed twice in 1× PBS at 4°C. Anti-rabbit Alexa Fluor 488 (Molecular Probes) with a dilution of 1:500 was used as a secondary antibody. Finally, the slides were washed twice in 1× PBS at 4°C, dehydrated in an ethanol series (70, 90, and 99%) at RT, mounted in Vectashield antifade (Vector Laboratories) with 10 μg/ml DAPI and covered with coverslips.

### Microscopy

Images were captured using an epifluorescence microscope BX61 (Olympus) equipped with a cooled CCD camera (Orca ER, Hamamatsu). Pseudocolors were applied using Adobe Photoshop CS6.

### Western Blotting Analysis

Nuclear proteins from young sorghum seedlings were isolated according to Gendrel et al. (2005) and Karimi-Ashtiyani et al. (2015). For Western detection, a 1:2,000 diluted SbCENH3 antibody in 1x PBC with 5% (w/v) low-fat milk was applied at 4°C for 12 h. Proteins bound by antibodies were detected with 1:5,000 diluted anti-rabbit antibodies 800CW (925-32213, Li-COR, Lincoln, NE, United States) for 1 h at 22°C. Fluorescence signals were recorded using Odyssey (Li-COR, Lincoln, NE, United States) as recommended by the manufacturer.

### CENH3 Chromatin Immunoprecipitation Sequencing

For nuclei isolation, 1 g of fresh leaf tissue was homogenized in liquid nitrogen and mixed with 10 ml of nuclei isolation buffer [1 M sucrose, 5 mM KCl, 5 mM MgCl_2_, 60 mM HEPES pH 8.0, 5 mM EDTA, 0.6% Triton X-100, 0.4 mM PMSF, 1 μM pepstatin A, cOmplete protease inhibitor cocktail (Roche)]. The nuclei were then fixed in 1% PFA in nuclei isolation buffer at RT and shaken at 12 rpm for 10 min. The cross-linking reaction was terminated by addition of glycine to a final concentration of 130 mM. The solution was filtrated through Miracloth (Millipore) twice and a 50-μm CellTrics filter (Sysmex) once and centrifuged at 4°C, 3,000 × *g* for 10 min. The pellet was resuspended in 1 ml extraction buffer [0.25 M sucrose, 10 mM Tris–HCl pH 8.0, 10 mM MgCl_2_, 1% Triton X-100, 1 mM EDTA, 5 mM β-mercaptoethanol, 0.1 mM PMSF, 1 μM pepstatin A, cOmplete protease inhibitor cocktail (Roche)], transferred to a 1.5 ml tube, and followed by centrifugation at 4°C, 12,000 × *g* for 10 min. The supernatant was removed and nuclei were resuspended in 100 μl nuclei lysis buffer [20 mM Tris–HCl pH 8.0, 10 mM EDTA, 1% SDS, 0.1 mM PMSF, 1 μM pepstatin A, cOmplete protease inhibitor cocktail (Roche)]. Chromatin was sonicated with a Bioruptor (Diagenode) using seven cycles of 30 s ON, 30 s OFF, for three times. The samples were then diluted 10 times with ChIP dilution buffer [16.7 mM Tris–HCl pH 8.0, 167 mM NaCl, 1.1% Triton X-100, 1 mM EDTA, cOmplete protease inhibitor cocktail (Roche)], centrifuged at 4°C, 13,000 × *g* for 5 min, and the supernatant was transferred to a 1.5 ml tube. The chromatin was mixed with 1:100 diluted SbCENH3 antibody and incubated at 4°C by shaking at 14 rpm for 12 h. Dynabeads^TM^ Protein A (Invitrogen) in ChIP dilution buffer, corresponding to one-tenth volume of the chromatin solution, was added to the antibody-prebound chromatin and incubated at 4°C by shaking at 14 rpm for 1.5 h. The tube was put on a magnetic stand and all liquid was removed after the solution was cleared. Beads were then washed twice with low salt buffer (150 mM NaCl, 0.1% SDS, 1% Triton X-100, 2 mM EDTA, 20 mM Tris–HCl pH 8.0), followed by two washes with high salt buffer (500 mM NaCl, 0.1% SDS, 1% Triton X-100, 2 mM EDTA, 20 mM Tris–HCl pH 8.0) at 4C by shaking at 14 rpm for 5 min. The bead-bound chromatin was purified by using iPure kit v2 (Diagenode) following the manual and quantified by using Qubit^TM^ dsDNA HS Assay kit (Invitrogen). The ChIP sequencing was performed using the NovaSeq 6000 system (Illumina) by Novogene (China), in the format of PE reads with 150 bp per end, and at least 6 GB raw PE reads were generated.

### ChIPseq Analysis

The reads of SbCENH3-ChIPseq and input-seq were quality checked and filtered as mentioned above, using tools implanted in the Galaxy-based RepeatExplorer (see text footnote 1) portal. ChIP-Seq Mapper (Galaxy version 0.1.1) (Neumann et al., 2012) was used to evaluate the enrichment of repetitive sequences in sequencing data from CENH3-ChIP experiments, with the repeat contig sequences of *S. bicolor* identified by RepeatExplorer as a reference.

## Results

### Satellite DNA Is Less Abundant but More Diverse Than LTR Repeats Among *Sorghum* Genomes

To study the genome divergence among *Sorghum* species, the repeat composition of five related accessions was analyzed. The cultivated diploid *S. bicolor* “V9” (SbV9) is an early flowering accession (Hsieh et al., 2015). The other two diploid accessions, *S. bicolor* ssp. *verticilliflorum* “WL” (SbWL), which is a wild relative to *S. bicolor* and *S. bicolor* var. *technicum* “YL” (SbYL) which is a cultivated species, are commonly seen as feral sorghums and are morphologically distinct from *S. bicolor*, especially in inflorescence architectures. The two tetraploid *S. halepense* accessions, “TT” (ShTT) and “US” (ShUS), are wild collections in Taiwan and the United States, respectively. First, the genome sizes of the diploid (2*n* = 2x = 20) and tetraploid (2*n* = 4x = 40) accessions were 789 ∼ 800 and 1,512 ∼ 1,547 Mb/1C, respectively, determined by flow cytometry (Table 1). In between the diploid and tetraploid accessions, we did not observe severe genome size differences.

Next, to compare the genome composition between different accessions, the high-copy repeat fractions were analyzed both individually and comparatively. Irrespective of the genotype, about 55% of the genomes are composed of moderate and high-copy repeat sequences (Table 2). Among them, the retrotransposon Ty3_gypsy is the most predominant component (36.5–39.3%), followed by either Ty1_copia (4.9–6.7%) or satellite sequences (4.7–5.8%), while DNA transposons and rDNA both account for less than 1.3%. Six different clades of Ty3_gypsy retrotransposons were identified, of which Athila is the most abundant in a range of 13.7–15.3%. Out of the eight detected Ty1_copia retrotransposon classes, SIRE is the most abundant one. Although Ty1_copia is not the most abundant, it seems to be the most diverse repeat type in all five *Sorghum* genomes.

**TABLE 2:** The proportion of moderate and high-copy repetitive DNA in the five *Sorghum* genomes.

**Repeats**		**Lineage/class**	**Clade**	**Genome proportion (%)**				
				**SbV9**	**SbWL**	**SbYL**	**ShTT**	**ShUS**
LTR retroelements	Ty1_copia	Ale		0.067	0.081	0.080	0.083	0.074
		Angela		0.089	0.081	0.050	0.107	0.111
		Bianca		0.093	0.117	0.110	0.211	0.089
		Ikeros		0.082	0.078	0.060	0.059	0.073
		Ivana		0.000	0.029	0.030	0.036	0.014
		SIRE		4.466	4.768	4.370	5.824	6.162
		TAR		0.184	0.223	0.210	0.246	0.242
		Tork		0.000	0.015	0.020	0.022	0.027
		*Total Ty1_copia*		4.981	*5.392*	*4.93*	*6.588*	*6.792*
	Ty3_gypsy	Non-chromovirus	Athila	14.023	14.972	13.710	14.275	15.300
			Ogre	5.739	6.045	5.670	4.899	5.090
			Retand	7.290	7.513	7.050	6.243	5.467
		Chromovirus	CRM	1.480	1.480	1.440	1.659	1.919
			Tekay	10.039	9.318	9.080	9.477	9.527
			Reina	0.016	0.014	0.020	0.036	0.016
		*Total Ty3_gypsy*		*38.587*	*39.342*	*36.97*	*36.589*	*37.319*
DNA transposons	TIR	EnSpm_CACTA		1.020	1.030	0.950	1.119	1.148
		MuDR_Mutator		0.140	0.125	0.110	0.084	0.124
	Helitron			0.000	0.027	0.000	0.000	0.000
		*Total DNA transposons*		*1.160*	*1.182*	*1.060*	*1.203*	*1.272*
LINE				0.000	0.000	0.000	0.013	0.011
Pararetrovirus				0.000	0.000	0.000	0.000	0.077
Tandem repeats	Satellite			5.870	5.169	5.580	5.656	4.799
	rDNA			0.873	1.174	1.210	1.043	1.137
Total annotated repeats				51.471	52.259	49.750	51.092	51.407
Unclassified repeats				4.018	4.573	4.930	3.584	4.837
Total repeats in analyzed clusters				55.489	56.832	54.680	54.676	56.244

*Italicized represent the sum of a subtype.*

**FIGURE 1:**
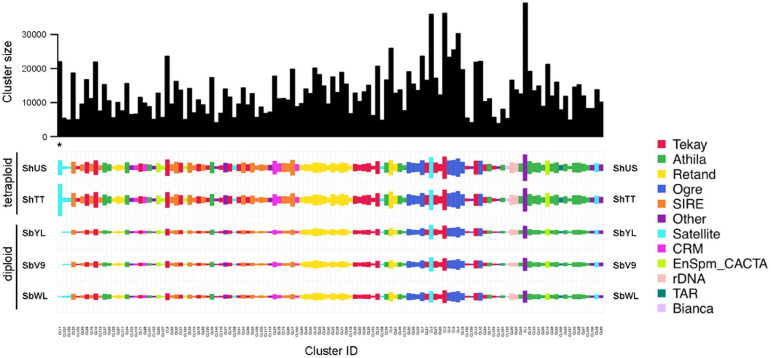
Comparative repeat analysis of the five *Sorghum* accessions. The top 141 clusters with a proportion greater than 0.1% of the analyzed reads are shown. The bar plot (top) shows the size (number of reads) of individual clusters. The color of rectangles represents the final annotation of each cluster, and the size is in proportion to the abundance of the repeat in each genome. The species and clusters are ordered using hierarchical clustering based on the correlation of quantities. The position of the satellite-type cluster CL11 (one of the two repeats with an enriched abundance in *S. halepense*) is marked with an asterisk.

To verify whether the high-copy repeats are shared or genotype-specific, a comparative RepeatExplorer analysis was performed using reads representing 0.1× genome coverage of each sample. The composition of the top 141 clusters with a proportion of more than 0.1% of the analyzed reads is shown in Figure 1. The read composition of satellite and LTR repeat-annotated clusters identified by TAREAN is listed in Table 3.

**TABLE 3:**
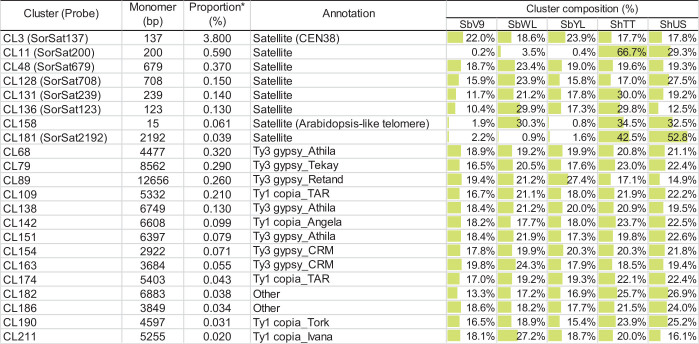
Cluster composition of the satellite and LTR repeats in the five *Sorghum* genomes.

*The proportion represents the percentage of read number in a cluster over the total analyzed sequence reads.

All high-copy clusters of retrotransposons were shared and almost equally enriched in all five *Sorghum* accessions. Nevertheless, the genome compositions of satellite repeats were variable among the accessions, specifically the satellite-type clusters CL11 and CL181, which are only abundant in the tetraploid *S. halepense* (Figure 1 and Table 3). The cluster CL11 with a monomer length of 200 bp was mainly represented by reads of ShTT (66.7%) and ShUS (29.3%), and only by a minor amount of SbWL (3.5%) as well as SbV9 and SbYL (less than 0.5% each) (Table 3). Similarly, over 95% of reads in the cluster CL181 were specific to the tetraploid *S. halepense* (42.5% from ShTT and 52.8% from ShUS). The satellite-annotated cluster CL158 with the monomer sequence (TTTAGGGTTTTAGGG), similar to the *Arabidopsis*-type telomere sequence, was enriched in SbWL (30.3%), ShTT (34.5%), and ShUS (32.5%) reads. In summary, most of the high-copy repeat clusters were shared and similarly abundant in the five genomes, only two satellite repeats (CL11 and CL181) showed a clear differential enrichment between the diploid and tetraploid genomes. In addition, CL158 was found to be more abundant in the tetraploid *S. halepense* and also in the wild diploid *S. bicolor* accession (SbWL).

Thus, although the total repeat frequency of all five *Sorghum* accessions is similar, the fast-evolving satellite repeats differ in copy number among genomes. These satellite repeats might play a key role in driving the diversification of the genomes, specifically distinguished diploid from tetraploid genomes.

### Comparative FISH of *Sorghum* Satellite Repeats Revealed Drastic Genome Rearrangements at Chromosomal Ends Subsequent to the *S. halepense* Formation

**FIGURE 2:**
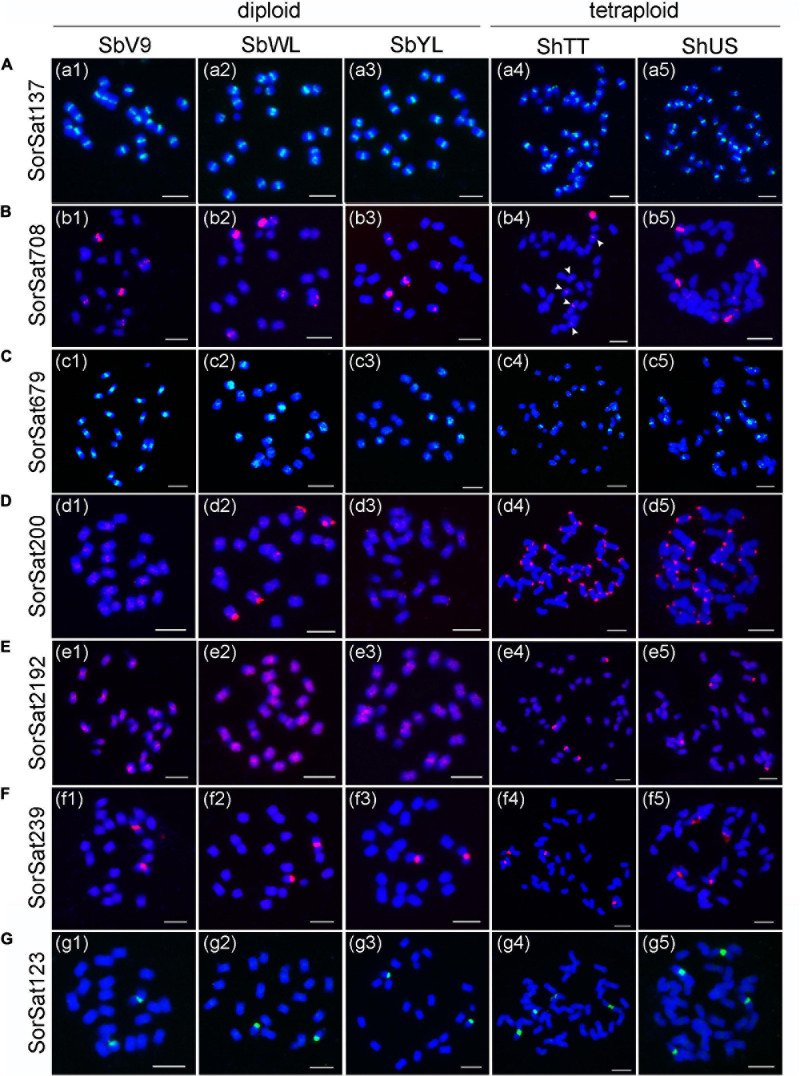
FISH mapping of the seven satellite repeats in the five *Sorghum* accessions. The satellite repeats include **(A)** SorSat137, **(B)** SorSat708, **(C)** SorSat679, **(D)** SorSat200, **(E)** SorSat2192, **(F)** SorSat239 and **(G)** SorSat123. Mitotic metaphase chromosomes were counterstained with DAPI. The signals of the satellite probes were pseudocolored in either red or green. The weak signals in (b4) are indicated by arrowheads. The name of *Sorghum* accession is labeled in abbreviation (Table 1). Bar = 5 μm.

Fluorescence *in situ* hybridization mapping of the seven high-copy satellite repeats, except for the *Arabidopsis* telomere-like CL158, which intermingles with the canonical plant telomeric repeats (TTTAGGG)_n_ (Supplementary Figure 1), was performed to elucidate their chromosomal distribution in all five *Sorghum* accessions. The corresponding FISH probes of individual satellite clusters were named based on their consensus monomer sizes (Table 3). SorSat137, representing the *Sorghum* centromeric repeat CEN38 (Zwick et al., 2000), revealed centromere-specific signals on all chromosomes of the three diploid accessions (Figures 2a1–3), as well as of the two *S. halepense* accessions (Figures 2a4,5). The satellite SorSat708 selectively accumulated in the pericentromeric regions of three chromosome pairs of the diploid accessions. While one chromosome pair showed very strong signals, the punctual signals on the other two pairs were severely weaker (Figures 2b1–3). In the two tetraploid *S. halepense* accessions, either eight signals (four strong and four weak) in ShUS (Figure 2b5) or five to six signals (one strong and four to five weak) in ShTT (Figure 2b4) were detected. In the latter case, obviously, a heteromorphic distribution of this satellite repeat occurred in at least one homologous chromosome pair. SorSat679 revealed signals in the pericentromeric regions of all SbV9 chromosomes, except for the smallest chromosome pair where this sequence was enriched in the telomeric region of one end (Figure 2c1). Nevertheless, SorSat679 displayed a relatively disperse distribution in the other accessions, with a preference in the pericentromeric regions (Figures 2c2–5).

SorSat200 was found to be accumulated at either or both ends of almost all *S. halepense* chromosomes, except three chromosome pairs on which no signals were detectable (Figures 2d4,5). In the diploid accessions, strong SorSat200 signals were only detected at the end of two chromosome pairs of SbWL (Figure 2d2), while the remaining chromosomes, including those of SbV9 and SbYL revealed only weak signals in the pericentromeric regions (Figures 2d1,3).

SorSat2192, the second satellite enriched in *S. halepense*, showed strong signals at the end of two chromosome pairs, in addition to weaker signals in pericentromeric regions in both tetraploid accessions (Figures 2e4,5). However, SorSat2192 resulted in only dispersed signals in the pericentromeric regions in diploid accessions (Figures 2e1–3). Thus, the chromosomal distributions of SorSat2192 and SorSat200 differ between the diploid and the tetraploid accessions. The satellites enriched in *S. halepense* tend to accumulate at the chromosome ends of *S. halepense*.

**FIGURE 3:**
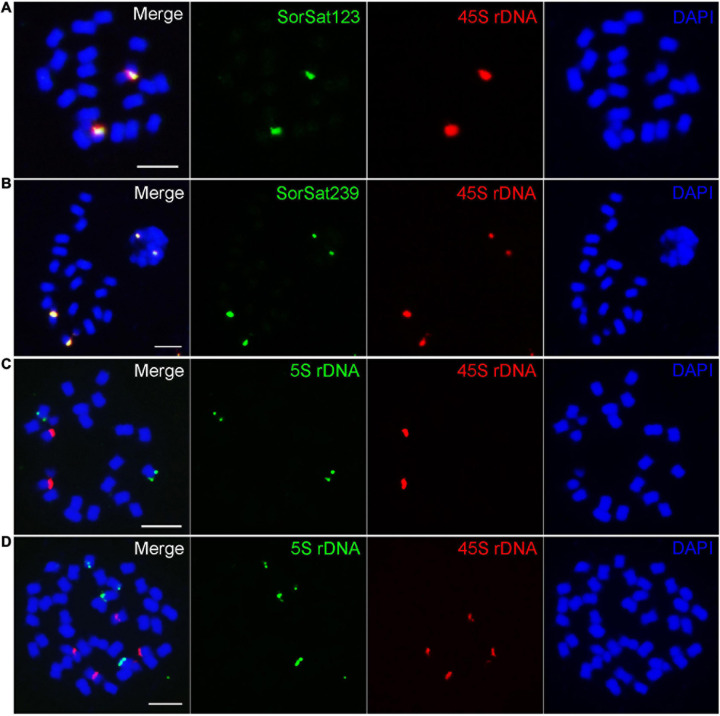
Fluorescence *in situ* hybridization mapping of the satellite repeats and rDNAs. The satellite **(A)** SorSat123 and **(B)** SorSat239 colocalize with 45S rDNA in metaphase chromosomes of *S. bicolor*. **(C)** One pair of each 5S and 45S rDNA signal is detected in diploid *S. bicolor*, while **(D)** two pairs of signals for both rDNA are present in tetraploid *S. halepense*. Mitotic metaphase chromosomes were counterstained with DAPI. Bar = 5 μm.

Both SorSat239 and SorSat123 showed signals on one chromosome pair in all diploid accessions (Figures 2f1–3, g1–3), and on two chromosome pairs in both tetraploids (Figures 2f4,5, g4,5). The colocalization of SorSat123 (Figure 3A) and SorSat239 signals (Figure 3B) with signals derived from a 45S rDNA-specific probe indicates the close proximity of both satellites to the nucleolus organizer region (NOR) in *S. bicolor* and *S. halepense* (signals in *S. bicolor* was shown as an example, Figures 3A,B). The 5S rDNA was detected on one and two 45S rDNA-negative chromosome pairs in diploid *S. bicolor* and tetraploid *S. halepense*, respectively (Figures 3C,D).

In summary, the chromosomal localization of the centromere-specific SorSat137, the NOR-associated SorSat123 and SorSat239 repeats is conserved in diploid *S. bicolor* and tetraploid *S. halepense*. The other four satellites SorSat200, SorSat679, SorSat708, and SorSat2192 showed distinct distribution patterns among accessions with respect to their loci number, copy number, and chromosomal localization. Especially, these satellites tend to locate in the pericentromeric regions of the diploid accessions, while the *S. halepense*-enriched satellites are particularly accumulated at the chromosome ends. The diversity in satellite distribution among the accessions indicates that satellite repeats might be involved in the process of genome diversification within the genus *Sorghum*.

### The Centromeric DNA of *S. bicolor* Is Composed Predominantly of a Single Satellite Repeat and Diverse LTR Sequences

**FIGURE 4:**
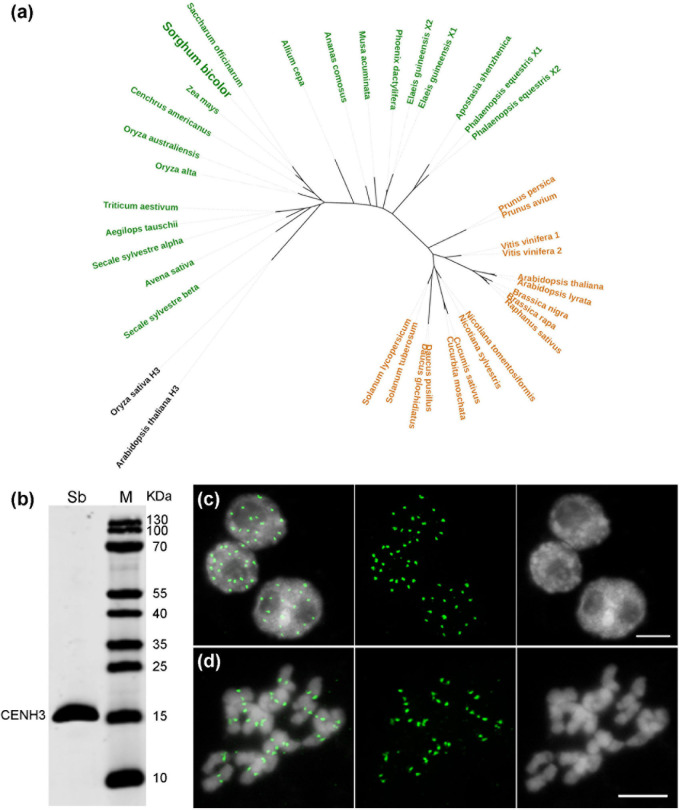
Phylogeny and antibody specificity of CENH3 of *S. bicolor*. **(a)** Phylogenetic relationship of CENH3 between *S. bicolor* and other plant species. The monocot and eudicot species are labeled in green and orange, respectively, and the canonical histone H3 used as outgroup are shown in black. The accession number of the CENH3 protein sequences are listed in Supplementary Table 1. **(b)** The specificity of an anti-SbCENH3 antibody probed on nuclear proteins of *S. bicolor* by Western blot analysis. Sb, *S. bicolor*. Immunodetection of SbCENH3 antibody shows distinct punctual signals (green) in interphase nuclei **(c)** and centromere-localized signals (green) in mitotic metaphase chromosomes **(d)**. Nuclei and chromosomes were counterstained with DAPI. Bar = 5 μm.

Although the centromere-localized satellite CEN38 was reported already before (Zwick et al., 2000), DNA sequences interacting with the CENH3-containing nucleosomes have not been determined so far in *Sorghum*. The *CENH3* gene of *S. bicolor* was identified from NCBI GenBank (Supplementary Table 1), and clusters phylogenetically with the CENH3s of the closely related monocots, sugarcane (*Saccharum officinarum*) and maize (*Zea mays*) (Figure 4a). To perform *Sorghum* CENH3-ChIP (chromatin immunoprecipitation), part of the N-terminal sequence was used for raising a peptide antibody (anti-SbCENH3) against the CENH3 protein. The specificity of the SbCENH3 antibody was confirmed by Western blot analysis, detecting a protein of the predicted size of 17.75 kDa (Figure 4b). Immunostaining with anti-SbCENH3 antibody resulted in distinct signals in interphase nuclei as they are typical for centromeres in species without Rabl orientation and in centromere-specific signals on metaphase chromosomes of *S. bicolor* (Figures 4c,d).

**FIGURE 5:**
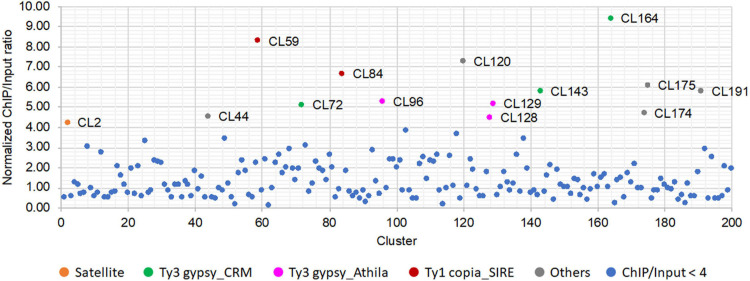
Relative enrichment of the repeat DNA clusters identified after CENH3-ChIP sequencing of *S. bicolor*. The enrichment ratio was calculated based on the read number of SbCENH3-ChIP DNA relative to the input DNA. The repeat clusters with ratio lower than four was labeled in blue and those with ratio higher than four was labeled in different colors according to their annotation.

After anti-SbCENH3 ChIP sequencing, 13.3 and 17.5 million of ChIP and input reads, respectively, were mapped to all the repeat clusters of *S. bicolor* using ChIP-Seq Mapper. The enrichment of individual repeat clusters was calculated and normalized according to the number of analyzed reads. In total, 14 clusters showed a higher ratio of ChIP/input than the threshold of four (Figure 5 and Table 4). In line with our FISH data, CL2 (SorSat137) is the only satellite repeat interacting with the centromeric nucleosomes (Figure 5). Eight LTR repeat clusters, including three Ty3_gypsy-CRM (CL72, CL143, and CL164), three Ty3_gypsy-Athila (CL96, CL128, and CL129), and two Ty1_copia-SIRE (CL59 and CL84), as well as five unclassified repeats (CL44, CL120, CL174, CL175, and CL191) were detected as centromeric candidate sequences (Table 4). Nevertheless, the genome abundances of these LTR repeats were in the range of 0.02–0.45%, at least 10 times less than the centromeric satellite repeat SorSat137 (4.7%) (Table 4).

**TABLE 4:** Centromere-associated repetitive sequences in *S. bicolor*.

**Cluster (probe)**	**Genome proportion (%)**	**ChIP/input ratio**	**Repeat annotation**
CL2 (SorSat137)	4.70	4.21	Satellite
CL44	0.45	4.50	Other
CL59 (SorSIRE_LTR1)	0.39	8.25	Ty1 copia_SIRE
CL72 (SorCRM_LTR2)	0.36	5.09	Ty3 gypsy_CRM
CL84	0.33	6.63	Ty1 copia_SIRE
CL96	0.29	5.25	Ty3 gypsy_Athila
CL120	0.21	7.25	Other
CL128	0.16	4.46	Ty3 gypsy_Athila
CL129	0.16	5.14	Ty3 gypsy_Athila
CL143 (SorCRM_LTR3)	0.08	5.76	Ty3 gypsy_CRM
CL164 (SorCRM_LTR4)	0.04	9.36	Ty3 gypsy_CRM
CL174	0.03	4.69	Other
CL175	0.03	6.06	Other
CL191	0.02	5.76	Other

To verify the centromeric enrichment of the repeats, four repeat clusters were selected for FISH mapping. Of them, one cluster, SorSIRE_LTR1 (CL59), belongs to the most abundant Ty1_copia lineage in this genome. The other three clusters, SorCRM_LTR2 (CL72), SorCRM_LTR3 (CL143), and SorCRM_LTR4 (CL164), were annotated as CRM (centromeric retrotransposon of maize) family, which is one of the centromere-associated transposon families commonly found in plants (Jiang et al., 2003; Neumann et al., 2011).

**FIGURE 6:**
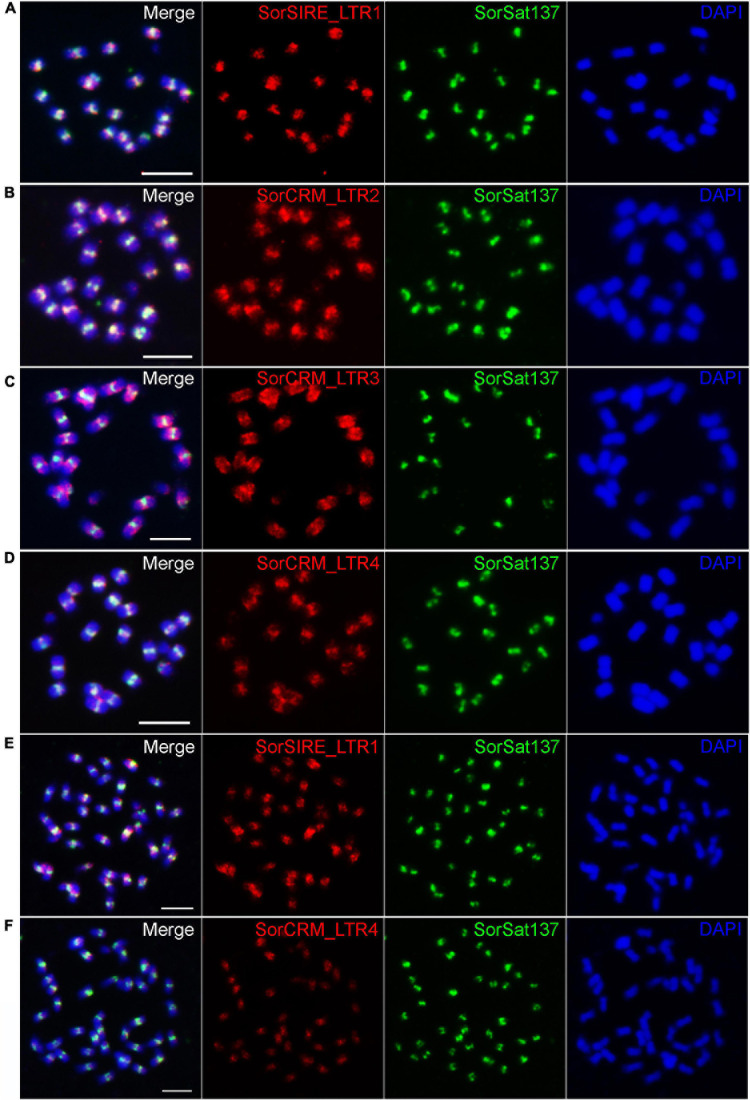
Fluorescence *in situ* hybridization mapping of centromeric repeats in *S. bicolor* and *S. halepense*. In *S. bicolor*, colocalization of **(A)** SorSIRE_LTR1, **(B)** SorCRM_LTR2, **(C)** SorCRM_LTR3, and **(D)** SorCRM_LTR4 with the centromeric satellite SorSat137, confirming the centromere enrichment of LTR repeats. The conserved centromeric distribution of **(E)** SorSIRE_LTR1 and **(F)** SorCRM_LTR4 in *S. halepense* is shown. Chromosomes were counterstained with DAPI. Bar = 5 μm.

All these four LTR repeats hybridized to the (peri)centromeric regions of *S. bicolor* (Figure 6), although not necessarily exclusively; for example, SorCRM_LTR3 showed a slightly more dispersed distribution in the centromere proximity (Figure 6C). The signals of the two LTRs with the highest ChIP/Input ratio (Table 4), SorSIRE_LTR1 (ChIP/Input ratio: 8.25) and SorCRM_LTR4 (ChIP/Input ratio: 9.36), overlapped strongly with the centromeric SorSat137 repeat (Figures 6A,D). The centromeric colocalization of the four tested LTRs with the predominant SorSat137 satellite indicated their association with *S. bicolor* centromeres, consistent with the CENH3-ChIPseq results. All these centromeric repeats seem to be conserved in the tetraploid *S. halepense*, as it is exemplarily shown for SorSat137 (Figures 2a4,5) as well as SorSIRE_LTR1 and SorCRM_LTR4 (Figures 6E,F).

## Discussion

### Divergence of Satellite Repeats Subsequent to *Sorghum* Polyploidization

Polyploidization has been long considered as an evolutionary event that leads to significant structural changes *via* genome rearrangement, especially after allopolyploidization when two distantly related genomes were combined (Otto, 2007). Many allopolyploids are superior to both parental species due to their beneficial characteristics in fitness and tolerance. The notably weedy *S. halepense* has been assumed to be an allotetraploid species formed by hybridization between *S. bicolor* and *S. propinquum* (Tang and Liang, 1988; Paterson et al., 1995, 2020). On the other hand, the rapid changes of fast-evolving satellite and retrotransposon DNAs alter genome composition and chromosome arrangement, leading to genome evolution. Incurred diversifying genomes pave the way for speciation. In this study, the genome-wide satellite and transposon categories and abundance were analyzed in five *Sorghum* accessions which were three diploid accessions, including two cultivated accessions (*S. bicolor*, *S. bicolor* var. *technicum*) and one weedy accession (*S. bicolor* ssp. *verticilliflorum*), and two tetraploid accessions of *S. halepense*.

The genome size of the tetraploid *S. halepense* accessions is around double of the *Sorghum* diploid accessions, with no genome downsizing detected in the polyploid genomes, which is similar to the example of Icelandic birch (Anamthawat-Jónsson et al., 2010). This phenomenon is opposed to the trend of genome downsizing among ancient angiosperm polyploids (Leitch and Bennett, 2004), likely indicating the recent origin of polyploidization in *Sorghum*.

The genomes of all *Sorghum* accessions comprise of about 55% of moderate and high-copy repeats, irrespective of the species or ploidy, demonstrating that no large-scale amplification or reduction of repeat sequences leading to a severe difference in total repeat content subsequent to the specification or polyploidization. Approximately 438 Mb of the *S. bicolor* genome is repetitive DNAs according to the RepeatExplorer-based analysis, which is very close to the estimated 460 Mb repeat fraction identified in the assembled genome of *S. bicolor* (Paterson et al., 2009). The comparison of genome-wide repeat profiles suggests that satellite DNAs played an important role in shaping the genome diversification in *Sorghum*, although their genome abundance (<6%) is much lower than that of the retroelements (>42%).

The chromosomal localization of the identified satellite DNAs revealed that drastic genome rearrangements might occur mainly in the *S. halepense* subtelomeric regions. Subtelomeres are known to be among the most dynamic and fast-evolving chromosomal regions (Contento et al., 2005; Garrido-Ramos, 2017) and often species-, genome-, or even chromosome-specific. For instance, diversification of subtelomeric satellite repeats between close *Leymus* species (Anamthawat-Jónsson et al., 2009) and between *Avena* subgenomes (Jiang et al., 2021) were reported. Subtelomeres are likely involved in facilitating homologous chromosome pairing during meiosis (Sadaie et al., 2003; Calderón et al., 2014) and play a role in maintaining chromosome ends and chromosomal stability (Mehrotra and Goyal, 2014; Padeken et al., 2015). In interspecific hybrids, pairing and recombination between non-homologous chromosome ends may lead to frequent sequence exchange and the birth of new subtelomeric repeats (Macas et al., 2006). The two satellite repeats enriched in *S. halepense*, SorSat200 and SorSat2192, are massively accumulated in the subtelomeres of *S. halepense*, while they preferentially reside in the pericentromeres in the diploid accessions. Hence, the accumulation of the two subtelomeric satellites, SorSat200 and SorSat2192, in the allotetraploid *S. halepense* might be a result of non-homologous recombination or large-scale genome rearrangement between pericentromeric and subtelomeric regions. Nevertheless, both satellite repeats are basically absent from the ends of *S. bicolor* chromosomes or at least only present at copy numbers not detectable by our FISH stringency. Additionally, SorSat200 shows similarity to the reported *S. halepense*-specific repeats XSR1 (64.7%), XSR3 (81.6%), and XSR6 (73.4%), which did not show any cross-reaction with the *S. bicolor* genome by Southern blot hybridization (Hoangtang et al., 1991). Hence, the accumulation of the satellite repeat(s) at the chromosomal ends must have happened after the speciation of *S. halepense*.

SorSat200, additionally, hybridized strongly at the four chromosome ends of diploid *S. bicolor* ssp. *verticilliflorum* (SbWL) but weakly in the pericentromeric regions of all three diploid accessions. Thus, SorSat200, although highly enriched in *S. halepense*, is not species-specific but exists in the diploid *Sorghum* species as well. SorSat200 might be a potential satellite DNA for evolutionary study of *Sorghum* genus due to its extraordinary diversity with respect to the chromosomal distribution.

Besides the disperse signals of SorSat2192 in the pericentromeric regions of all analyzed *Sorghum* accessions, selective accumulation at distal regions of two chromosome pairs was detected in the tetraploid *S. halepense*. A similar heterogeneous chromosomal distribution was found for the satellite *Khipu* in *Phaseolus vulgaris* (Richard et al., 2013). *Khipu* repeats were detected in both subtelomeres and centromeres, and even different copy numbers and sequence variants were present among chromosome ends. The availability of an assembled genome of *S. halepense* might help to understand the heterogeneous distribution of subtelomeric satellites in *Sorghum*.

In addition to the subtelomeric repeats, the satellite repeats SorSat679 and SorSat708, which preferentially locate in the pericentromeres, displayed chromosome-specific amplification in an accession-specific manner. Depending on the accession, different numbers of chromosomes showed enrichment of these repeats at distinct loci. Particularly, for SorSat708 a heteromorphic pattern on one chromosome pair of *S. halepense* “TT” was observed. Such heteromorphic distributions of repeat DNAs between homologous chromosomes were reported in other plant species, such as *Allium subvillosum* (Jamilena et al., 1990), *Brachycome dichromosomatica* (Houben et al., 2000), *Secale cereale* (Viinikka and Kavander, 1986), and *Triticum araraticum* (Badaeva et al., 1994). SorSat708 might reflect repeat composition diversity within the species *S. halepense*.

In contrast to the polymorphic distributions of pericentromeric and subtelomeric satellite repeats, the chromosomal association of SorSat123 and SorSat239 with 45S rDNA are conserved among the diploid and tetraploid *Sorghum* accessions. These repeats are likely originated from the intergenic 45S rDNA regions as also described for satellite repeats in other species (Macas et al., 2003; Garrido-Ramos, 2015) and might arose before the diversification of the studied *Sorghum* species.

The analysis of genome-wide repetitive profiles in the *Sorghum* accessions demonstrates the importance of satellite DNAs in shaping the genome divergence in *Sorghum*. In diploid *Sorghum* genomes, especially the differential amplifications of the pericentromeric satellite repeats, such as SorSat679 and SorSat708, among chromosomes contributed to their diversification. Among *Sorghum* genotypes, the observed polymorphic distribution of the subtelomeric satellites enriched in the tetraploids indicates drastic genome rearrangements after the allopolyploidization event forming *S. halepense*.

### The Sequence Composition of the Satellite-Dominated Centromere in *S. bicolor* Is Conserved in *Eusorghum*

Our CENH3 ChIP-seq analysis revealed that the centromeres of *S. bicolor* are mainly comprised of the satellite repeat SorSat137 (CEN38, Miller et al., 1998b; Zwick et al., 2000) with a genome proportion of 4.7% in combination with diverse retroelements. These retroelements include CRM as well as Athila of Ty3_gypsy and Ty1_copia-SIRE sequences with a total genome proportion of only 1.81%.

A comparison of the consensus sequences of the SorSat137 family members in the five *Eusorghum* genomes revealed a similarity of more than 92%. This repeat also exists in most centromeres of *S. officinarum* × *spontaneum* but with a lower sequence homology (Zwick et al., 2000), as well as in the *Sorghum* subgenera: *Chaetosorghum*, *Heterosorghum*, *Parasorghum*, and *Stiposorghum* (Anderson, 2005). Most likely, SorSat137 (CEN38, Miller et al., 1998b; Zwick et al., 2000) preexisted in the common ancestor of *Sorghum* and *Saccharum* and diverged during the evolution of *Sorghum* except in the subgenus *Eusorghum*.

The similarity of the centromeric composition of *Sorghum* and *Saccharum* is not restricted to the satellite repeat SorSat137. The centromeric Ty3_gypsy-CRM retroelements were also identified in the wild *Saccharum* species, *S. spontaneum* (Zhang et al., 2017). While Ty3_gypsy-CRM retroelements are commonly found in plant centromeres (Miller et al., 1998a; Neumann et al., 2011), Ty3_gypsy-Athila elements are less frequently detected in centromeric regions except in the centromere core of *Arabidopsis* (Kumekawa et al., 2000), *Festuca*, and *Lolium* species (Zwyrtkova et al., 2020).

The other *S. bicolor* CENH3 nucleosome interacting retroelement belongs to the SIRE class (or Sirevirus), which is the most abundant Ty1_copia retrotransposon of 4.5% in this genome. In most plant species, SIRE retrotransposons tend to show a disperse distribution along chromosomes or are preferentially enriched in pericentromeric heterochromatin (Weber et al., 2010; de Souza et al., 2018).

The distribution patterns of the identified centromeric satellite and retrotransposon repeats in the allotetraploid *S. halepense* are similar to those in the diploid *S. bicolor*. This high similarity in the centromeric composition implies the close relationship of both species and supports the assumption that *S. bicolor* might have been involved in the formation of *S. halepense*.

## Data Availability Statement

The data presented in the study are deposited in the European Nucleotide Archive (ENA) repository, accession number PRJEB46549 (http://www.ebi.ac.uk/ena/data/view/PRJEB46549).

## Author Contributions

Y-TK performed DNA extraction, repeat analysis, FISH, ChIPseq experiments, phylogenetic analysis, and wrote the manuscript. TI identified CENH3, performed western blot, and immunostaining. JF measured the genome size. W-HH collected the plant materials. AH and Y-RL designed the research. Y-TK, JF, Y-RL, and AH revised the manuscript. All authors contributed to the article and approved the submitted version.

## Conflict of Interest

The authors declare that the research was conducted in the absence of any commercial or financial relationships that could be construed as a potential conflict of interest.

## Publisher’s Note

All claims expressed in this article are solely those of the authors and do not necessarily represent those of their affiliated organizations, or those of the publisher, the editors and the reviewers. Any product that may be evaluated in this article, or claim that may be made by its manufacturer, is not guaranteed or endorsed by the publisher.

## Footnotes

1
https://repeatexplorer-elixir.cerit-sc.cz/galaxy/

2
http://iqtree.cibiv.univie.ac.at

3
https://itol.embl.de/

4
https://www.ncbi.nlm.nih.gov/Structure/cdd/wrpsb.cgi

5
www.lifetein.com
