# Supplementary material for: The Evolutionary Dynamics of Repetitive DNA and Its Impact on the Genome Diversification in the Genus Sorghum
Source: Front Plant Sci. 2021 Aug 12;12:729734. doi: 10.3389/fpls.2021.729734 (PMC8407070; doi:10.3389/fpls.2021.729734)
Supplement: Supplementary file 1 [file Data_Sheet_1.pdf]

Supplementary Table 1 NCBI accession numbers of the plant CENH3 protein sequences used in the phylogenetic analysis

NCBI accession number	Plant histone CENH3 or H3
AAA32809	*Arabidopsis thaliana* H3
ADI87407	*Oryza sativa* H3
XP 002441290	*Sorghum bicolor*
BAP26970	*Cenchrus americanus*
BAQ19382	*Avena sativa*
ANC33050	*Saccharum officinarum*
XP 010931498	*Elaeis guineensis* X2
XP 019708718	*Elaeis guineensis* X1
XP 008792454	*Phoenix dactylifera*
AKI32604	*Musa acuminata*
XP 020113817	*Ananas comosus*
BAL45432	*Allium cepa*
ACX30889	*Oryza alta*
ACX30893	*Oryza australiensis*
AKM28569	*Aegilops tauschii*
AEH95350	*Triticum aestivum*
NP 001105520	*Zea mays*
XP 020572267	*Phalaenopsis equestris* X1
XP 020572268	*Phalaenopsis equestris* X2
ACZ04978	*Brassica nigra*
NP 001288957	*Brassica rapa*
BAF49733	*Raphanus sativus*
AAL86775	*Arabidopsis thaliana*
AAT96392	*Arabidopsis lyrata*
NP 001289450	*Nicotiana tomentosiformis*
NP 001289496	*Nicotiana sylvestris*
AID21730	*Daucus pusillus*
AID21731	*Daucus glochidiatus*
XP 007219168	*Prunus persica*
XP 021815253	*Prunus avium*
XP 006339687	*Solanum tuberosum*
XP 010326926	*Solanum lycopersicum*
XP 010661899	*Vitis vinifera* 1
XP 002281073	*Vitis vinifera* 2
XP 011659153	*Cucumis sativus*
XP 022959605	*Cucurbita moschata*
AUN88469	*Secale sylvestre beta*
AUN88454	*Secale sylvestre alpha*
PKA51165	*Apostasia shenzhenica*

Supplementary Table 2 Primers used for PCR amplification and FISH probe generation

Probe (cluster)	Forward primer (5'-3')	Reverse primer (5'-3')	Template DNA
SorSat137 (CL3)	F: GCTAAGAAATCATTTTGGACGCA	R: CATTAGGTGCAAACCGTGCA	SbV9
SorSat200 (CL11)	F: CACCTCGAGGGACCCTGTC	R: CCCTCTAGCTATGAACTATTGGT	ShTT
SorSat679 (CL48)	F: GAACTCATAGCCACGCAGC	R: CTAGGGTTCGGTCAAAGGGT	SbV9
SorSat708 (CL128)	F: GTCCGTTCGTAAAACAGCCA	R: CGGATGGGTTGTAGGACAT	SbV9
SorSat239 (CL131)	F: ATTACGTGGATTTTGGGGCC	R: AGTGTTTTCCTGCAGTTTCACC	ShTT
SorSat123 (CL136)	F: CTCACGCCTTGCCGAACT	R: CACGTGGTCTGTGCGCTG	SbWL
SorSat2192 (CL181)	F: GAGGCATGCAGAAGTGATGG	R: GAGCACCGTGACTCAGAGAAG	ShTT
SorSIRE_LTR1	F: CAAGCATTGGTGGAAACAAA	R: TGCCAAATTCATCACAAAGC	SbV9
SorCRM_LTR2	F: CCCCAATTTGAACCACAAAG	R: GTCAAAAGGCAAAGTCACG	SbV9
SorCRM_LTR3	F: TTTGTGGGTGACAAGTGGTG	R: GGGGAGGGACTCTATCTTCG	SbV9
SorCRM_LTR4	F: TCTAATCCACATCGCAGCAC	R: CCTCAAACATGGAAAGATGTGA	SbV9

Supplementary Table 3 Consensus sequence of the repeat clusters used for primer design. Primer sequence positions are indicated in ***bold***.

Probe (cluster)	Consensus sequence (5´-3´) *
SorSat137<br>(CL3)	>SoSat137_CL3<br>G**TGCACGGTTTGCACCTAATG**CACCATAT**GCTAAGAAATCATTTTGG**<br>**ACGCA**CCCGATGGAACTCCTAGATGAAGTGTCTCATATGGAATCTCG<br>CTTCGGTCTGTTTGGAGATAGTGCTAATTTTGGTGCAAGATAG
SorSat200<br>(CL11)	>SorSat200_CL11<br>GCTTGAGG**ACCAATAGTTCATAGCTAGAGGG**ACCCCATCA**CACCTC**<br>**GAGGGACCCTGTC**ACAGCTCGAGATAGCCTATCACAGCTGAAAAAG<br>TCTAGGACTCCAAACCATGGTAAAATTCTAGACTTTTCCATACTTTTAT<br>GTGTCCAAACCATAGTAAAATTCCATGTGTTCAAGGTATGGTAATATT<br>TTTTTGTGAAA
SorSat679<br>(CL48)	>SorSat679_CL48<br>TTGAATTTTCATA**GAACTCATAGCCACGCAGC**GGTTTCGGAACCCTA<br>GATGGTCTCGAATGGAAAAGTCATGAATACCAATATTGTTCCACTCAT<br>CAAAATCTACATTTAATATATAGACCATTTTTGCATTTGACAAAGTGTT<br>GGGAAGGTGTAGTTGAAAATCCACAATTGACACATATAGTTTCATATA<br>GTTTGTGTGAGATTCAAGATTTGGTGGGTATTGTTAACTTAAACTTTC<br>TCAAATGGAAAAATTGTCTATATAAAAAGTTTAGATCTTGATGATATCT<br>AACAACTTGGTATTCAAAATTTTTTCATTTTAAGTAATTTAGTTTGTCAT<br>TTGACCAAGTTTGACCAAAACCCGTCTTGGATTTTAAACAAATGTGCT<br>TAGGATTGGCTCAAATTTATCCAAATGGAAAAATGGACAATATATCAA<br>ATGTAGATCTTGATGATCTCTAACAACTTTGTATTCAAAATTTTTTCAT<br>TTGAAGTCATCAAATGGGTCATTTGACCAAGTTTGACCAAAGTCAAAG<br>CATTGGTTTTTACAAACAC**ACCCTTTGACCGAACCCTAG**ATGGTCCC<br>AAATGGAAAAGTCATGAATACAAAGTTTGTTACACTCATCAAGTTCTA<br>CATTTTGTCTAATGGTCAACTTTTCATTTGGAAAAGTTTGAACCACTG<br>AATT
SorSat708<br>(CL128)	>SorSat708_CL128<br>GTTTCCATCAATTTCTAGCCACCACCGCAAATTTTTCCCACTTCCGGA<br>CCGTTTTGTCCTCTAATTCGGA**GTCCGTTCGTAAAACAGCCA**TAACT<br>TTCGCATACGAACTCCGATTTTGACGTTCCATATATCAAAATCGATCA<br>GAAAAAATTTTTGGATCTGTCTATCCCCCCCAACGGCGGGTTCGACA<br>ATTATAGGATCCCCAAATTCGCCTCTGAAGTTTGAGTTTTCACCTCCT<br>AAAGTTTTTTTTCATATTTTTCACAATCTTCAAAAAATTCCACAGGCCA<br>CGTCTTTCACTTGTTTAAGCTCAAATTTCCTGTGGTTACTTCTTGAGT<br>GCTCCTAAGTAAAAGAAAAATATCAAAAAAAAGAATTGTAGGGTTTCG<br>AATTGTGTTGTTCGCAAGCTGGATTGATATGTCTCTCGTTTCCACCAA<br>ATCGATAGTTACCAGAACCTTTCGGAAGATCGGGCCACACTTGTTCT<br>CCTCAAGTGGTAATCAAAGCTTCAGACATACCGTTAGGTTCACATCTA<br>CCCTTAGTTTCGAGTGTCCTTGTGTTCGTCCCATAAGTCTAGTGCCAT<br>ATTGTTTTC**ATGTCCTACAACCCATCCG**CGCCATAGCCTTTGCATATT<br>TCAGTTTCAGAGTCCGTAGTGTTGAGTTTGTGTCCTGGTCGAGGTCT<br>TGTTACTGGTTAGGTCTTGTTACAGTCCAGTTTTTTTCCCACT
SorSat239<br>(CL131)	>SorSat239_CL131<br>GTTTTTCGAGCTAACGAAAGCCGAACCCGGGGCGGCAGTCGCGCGA<br>CCGAACCTGCGTTTTTGGGCAAAAACCACATGGAATCTGCAT**ATTAC**<br>**GTGGATTTTGGGGCC**TTTTCAAAAACGCAGTAGAGAACCGTACGAAA<br>AATCTGCAGAACTTCTGGGGGGCAGGGATGCCCCTCTGGTACTATA<br>GGGGAGAGGGTTCGGCTGGCTGAGACCATG**GGTGAAACTGCAGGA**<br>**AAACACT**A
SorSat123<br>(CL136)	>SorSat123_CL136<br>TGGGCA**CTCACGCCTTGCCGAACT**GCTGTTTTTGCCCCCTGCCACTA<br>TATTCGTCACGTGAACGAAAGTCGGGTTTCCCGGGAATTCGG**CAGC**

	**GCACAGACCACGTG**CGCGCGCCACGAATTC
SorSat2192 (CL181)	>SorSat2192_CL181<br>CACGGGCGAAATTGGTTGCATATCATTGAGTGTCATAACGCATTACC<br>CGAACAAAACATTGCTCCCTCGTAAACTGACCATAAGGAAA**GAGGCA**<br>**TGCAGAAGTGATGG**CCATGCTATCTAGATCATATGCAATAATGTAAG<br>TAGTTGTTTTGAGGCCTTGTTTTACAAAGTAACGCTTCCCTACTTGTC<br>CCTAGCAATTTGGTACATAGGGAAGTAATTTCAAACACTATTTGTACT<br>AATGTTTGATTTCTCATCGGTTTGAATGCTTTCCATCGTAGAGAAACA<br>TTCGTGTATTTTGTCAACCATGCATCTAGTAACGCTTCAATTTTCTTAT<br>GCCCACTCTATCGGTTGCATTCAACCATCAGTACTAAACTAATCAATA<br>AAATAAGGCAACCATGTCATTAGTTAACGACAAGGATATATCCTTAGG<br>AATACGGGAATAGTGGACACTATGGAAATTTCATTTGCAAAAGTGGTT<br>TTGTGCTTTTACTATACTAAGTGTTGACCATCAGTCATTTTTCGCATG<br>CGAATAACAAATATAGCCATCGGAACAATAAATCAGTTGCTAATGCAC<br>CCAATCTAGGAAATTATTTAATGTTAATGCGCACAGATCCTTTCACAA<br>AATTGTATACCCATACAGAAAACTACATGTGCACTGAAAGTATTACAA<br>GGAAAGACACAAATAATCAACCACTGCTAAAGGCTAGCATATTTTTTA<br>ATTAATAGAATTTATTACAACAATAATTTCATTGACAATGGAAAGAACT<br>ACCAATTGTTATCAGCCAACTTGATAGTCCAGCTACAAAGATTTATAA<br>AAGACATGACATTACAAAGATTGCATGGGGCTTCGCTGTCAAGCGGT<br>TCACCTTGGGCAAAAATATGGTTGTTGATGTTGGGTTGACACATCAC<br>CCATGTGAGGGTGGTCTATTGGTTGTGGTTGGATAATACTTTGGGCA<br>TGCCTCCCTAAAGATTCTGACCCTGTTTGATCTGTGACAACCTACCCT<br>CATACTTCTCACGGCATTTCTCTTCACACGAGTTGAAGGAGGATTGG<br>TCATCCTTTAGCTTCTCGCAGTTCTGACGGCAGGCACCTACACCGAT<br>GGTCGCATAACTGAAGTGCTCGGTGAGGCTACACTTCTCCTTGCAAC<br>GGTCGATGTCGGCAGGCGTCCCCTTACCCTCACATTGCTTATTGCAG<br>ACATCTGCCTTGGCAACTACCGTGCTGACCTTGTCAGCAATTTCTCC<br>TGCTAGCACACATGGGGCTCCAAGGATGAGGGTCACTATGAATAGA<br>GCTCCAACAATGTTTCTCACCTGGGCCATTATTGTTGTCCCTATTCAG<br>CTATTGGATGATGAGACGGGTTTACTCATGGAGCTACTGTGTCATCC<br>TTTATATATAGTGGAGGGGAGGACATAAATTAGGCAAGGGTGTGAGG<br>CTACGAGAGCTTCACGCTGAACCATTTGGTGGTATGTGGATGCCCGT<br>CTTCCAATTTTCTTTGTCTTTCACTTTACCTCATAGGTGTTGTTCACTT<br>GGCAACCATGCATGGGTTTCAACCATTTTGGTTGGTGGAAACAAATT<br>GCCAATGCTTAGTAGGAAGTAGTAGTTTATTGTTGGCAACAATGTATG<br>GGTTTAGAACTTTTTTGGTATATCCGTAAACCAAACCATCATTGCCTG<br>TGCTAGGCTCCTTGTCCAATAAATATTAACCATCAATCTTCCAAAATA<br>AATTTTTGCACGTGTAGCCTATGATAGTCATCTCAATCTTTGTTCTGC<br>TCCTAAACCATTGCTATCACCTTTTATGTATTCACCCTATGATTTGATT<br>GACCTCTTCTATATTTGGTTAGGGTTGGGGTTCACAAGGGCAATAGT<br>AGCTCAATATCATGACAACAACTGCTCTAGTCATAAGAAATCCAGTTT<br>CTCCTCATGAAAAGACAATTTGAGAATTAATTAAAACAAAGGATTATT<br>GTTTGAGCACTTTTCATTAAACTTATACTAACAAGTTAACTAATTCACT<br>TTTAATAAGCACATGATGCTTCCGCATGTTGGATAAGTGCTCCATATT<br>TGAGTTTTTC**CTTCTCTGAGTCACGGTGCTC**TTAGCTCGTTCTAGGG<br>TAACACACTAAAGTGAAAGTAGTATTGGGCATTACCCATGTGGGCTA<br>ACATGTTTCTCTCCACCTAATGATCTTCCACTATCTCCGAACTTTAG
SorSIRE_LTR1 (CL59)	>SorSIRE_LTR1_CL59<br>GAGTTATTGCACATGGATTTATTTGGACCAACCACATACA**CAAGCATT**<br>**GGTGGAAACAAA**TATGGATTTGTGATAGTGGATGATTTCACAAGATA<br>CACATGGGTATTCTTTCTTAGTGACAAGAGTGATGCTTTTGCAACCTT<br>CAAATCATTTGTCAAGAGAATACACAATGAGTTTGAAACAACCATCAA<br>GAAAGTGAGAAGTGACAATGGAAGTGAATTCAAGAATACAAGAGTTG<br>ATGA**GCTTTGTGATGAATTTGGCA**TTAGGCATCAATTCTCGGCCAAG<br>TACACTCCACAATCAAATGGTCTTGTTGAGAGGAAGAATAGAACCTT

	GATTGACATGGCAAGATCAATGTTGAGTGAATACAATGTGAGTCATTC<br>TTTTTGGGCCGAAGCAATCAACACGGCTTGCTACTATAGCAACCGAC<br>TCTATTGTCACCCAATGATGGAGAAGACACCATATGAGCTCTTGAAT<br>GGAAGAAAGCCCAACATTGCATACTTTCGGGTTTTTGGTTGCAAATG<br>CTACATATTGAAGAAAGGCACTAGATTGAGTAAATTTGAAAAGAAATG<br>TGATGAAGGCTTCTTGCTTGGTTACTCCACTACTAGCAAAGCTTATAG<br>AGTTTGGAATTTGGCTAGTGGTACTCTTGAAGAGGTGCATGATGTTG<br>AGTTTGATGAAACCAATGGCTCTCAAGAGGAAGATGAGAATCTAGAT<br>GATGTGAGAGGCACTCAATTGGCCGATGCAATGAAGAATATGGATAT<br>TGGTGATTTAAGGCCTAGAGAGGTGATTGATGTTGAAGATGACAAAG<br>ATCAAGTGCTTCCTACCTCTAATGTGCAAGCTAGTGGTTCTCATGATC<br>AAAATCAAGCTAGTACAAGTGGTACACAAGTGCAAGATCAACAAGCT<br>AGTACATCATCTCATGATCAACCAAGTGCAAGCAATCAAGTGCAAATA<br>CTCCAACCAACAAATATTGCAAGAGATCATCCATTGGATCATATCATA<br>GGTGATATTCAAAGAGGAGTGCAAACTAGATCAAGATTAGCATCATTT<br>TGTGAGCATTTCTCCTTTGTGTCTCACATTGAGCCAAAGAAGATTGAT<br>GAAGCATTGAGAGATGTTGATTGGGTTAATGCTATGCATGAAGAGCT<br>TAACAATTTCAAGAGAAATCAAGTATGGGAATTAGTTGAGAGGCCTA<br>GTGATCATAATGTCATTGGTACTAGATGGGTCTTTCGCAACAAGCAA<br>GATCAAGATGGGATAGTTGTAAGGAACAAAGCAAGATTGGTAGCACA<br>AGGCTATACTCAAGTTGAAGGTCTTGACTTTGGTGAAACATATGCCC<br>CGGTTGCAAGATTGGAAGCAATTAGGATCTTGTTGGCCTATGCTTGT<br>GCTCATAACATCAAGCTATACCAAATGGATGTGAAGAGTGCATTTCTC<br>AATGGCTATATCAATGAAGAAGTTTATGTTGAGCAACCTCCCGGTTTT<br>GAAGATGACAAGAAACCCAACCATGTTTACAAGCTAAGAAAGGCATT<br>GTATGGTTTGAAGCAAGCACCTAGAGCATGGTATGAGAGATTGAGAG<br>ATTTCTTACTCTCTAAAGGTTTCAAGATGGGCAAGGTTGACACCACTC<br>TCTTCACCAAGAAGATTGGCAAAGACTTGTTTGTGTTGCAAATATATG<br>TTGATGATATCATATTTGGATCAACCAACCAAGAATTTTGTGAGGAGT<br>TTGGAAACATGATGGCTAATGAGTTTGAGATGTCAATGATTGGAGAG<br>CTTAGTTACTTCCTTGGTCTTCAAATCAAGCAATTGAAGAATGGCACA<br>TTTGTGAGTCAAGGCAAGTACATAAAAGACATGCTCAAGAAGTTTGG<br>TATGGATGATGCAAAATCAATTAGCACTCCAATGGGAACAAATGGAA<br>GCCTAGATAGTGATACAAGTGGAAATATGGTGGATCAAAAGTTGTAT<br>CGGTCTATGATTGGAAGCCTACTCTATGTGACCGCATCAAGACCGGA<br>TGTCATGTTTAGTGTATGCATGTGTGCAAGATTCCAAGCCTCACCAA<br>GAGAAAGTCATTTGAAAGCAACAAAGAGAATATTGAGGTACTTGAAG<br>CATACACAAAATGTTGGTTTGTGGTATCCCAAAGGTGCAAAGTTTGAA<br>CTTGTTGGATATTCCGATTCGGACTATGCGGGATGCAAAGTTGAGAG<br>AAGAAGCACATCGGGCACATGTCAACTATTGGGAAGATCACTTGTCT<br>CATGGTCATCCAAGAAGCAAAATAGTGTTGCACTATCAACCGCCGAA<br>GCGGAGTACATTGCGGCCGGTAGTTGTTGTGCACAAATCCTATGGAT<br>GAAGGCAACATTGAAGGACTTTGGAATCAATTTCAAACAAGTGCCATT<br>GCTATGTGACAATGAAAGTGCCGTGAAGCTCACCAACAATCCGGTTC<br>AACACTCAAGAACAAAGCACATAGATGTCCGCCATCACTTCATAAGA<br>GATCACCAACAAAAAGGGGACATTTGCATTGAGAGTATAGGCACCGA<br>TGATCAACTTGCCGATATATTCACCAAGCCACTTGATGAGAAGAGGT<br>TTTGCAAGCTAAGGAATGAATTGAACATACTTGACTTCTCAAATATGT<br>GTTGATGCACCCCCACTATATGACATGCCTCTCCTTCGAGCAAAGCA<br>AGGTAAAATTGTTTGACATGTCATCCATCCTATGCTAAGGACTTGTTT<br>AGTGCATCTAGTCATTCCTTACATGTCTTAGGCTCATTCATGAAAATC<br>AAATGAATTTGATGCTTGTATGGTACCACTATTGCTTCTATGCTTGAC<br>TTGATCTAGTGGTAGCATATGACATGTTTGTGGGCTTGCAATCCTAGT<br>GTTTGATCTAGAATATGAGCTATAAGTGTTTAACTCAACATGGTACAA<br>GATAACCCTTATTTGGAGGTGTGAAGAAGCTTGTCCTTGGATCAAAC<br>CGAGTTAAATATCTTAGGCAAGTAATCTAGATTGGACCAATTTGGGAA

	AATGATCTCACTTCACATGGTTTCACACTAACCTATCTAAAATTTGAG<br>CTCACCTTTTGTGGTCATTGATGACAAAGGGGGAGAAATTTTACGAG<br>AAGTACAAAGATAGGGGAGTAACATAATAAAAGAAGGGGATCGATTA<br>AAATTTTTGAAGCACACAAG
SorCRM_LTR2<br>(CL72)	>SorCRM_LTR2_CL72<br>GTAGTAGAATGCAATGAACGATTATAGGCGAACTCAATATGAGGCAA<br>ACATTCTTCCCACATCTTAATATTCTTCTTTAAAACAGCCCTTAACATA<br>GTGGATAAAGTTCTATTAACAACTTCAGTTTGACCATCAGTTTGGGGA<br>TGACAAGTGGTGGAAAATAAAAGCTTAGT**CCCCAATTTGAACCACAA**<br>**AG**TCTTCCAAAAATGACTAAGAAATTTAGCATCACGATCAGAAACAAT<br>TGTGTTGGGCACACCATGTAAGCGAACAATTTCTCGAAAGAACAAAT<br>CAGCAATATTTGTAGCATCATCAGTTTTATGACATGGTATGAAATGTG<br>CCATCTTAGAAAATCTATCAACAACCACAAACACACTATCACGTCCCC<br>TCCTAGTCCTTGGTAGTCCCAACACAAAATCCATAGATATATCCTCCC<br>AAGGAGCATTAGGAACAGGAAGAGGTAAATACAAACCGTGGGGATTT<br>AAC**CGTGACTTTGCCTTTTGAC**ATGTTGTGCAACGAGCAACAAACCT<br>CTCCACATCTCTCTTCATCCTTGGCCAAAAGAAATGACCAGCAAGTAT<br>GTCCTCGGTCTTCTTTGCTCCAAAATGTCCCATCAAGCCACCTCCAT<br>GCGCTTCCTGCAACAACAACAAACGAACAGAGCTAGCTGGAATGCAT<br>AGCTTGTTAGCTCTAAACACAAACCCATCACTAACGATGAATTTATTC<br>CACCCTTTTCCATCTTTACAATGCAGCAACACGTCTCTAAAATCAGCA<br>TCATGAACATATTGGTCTTTAATTGTTTCTAACCCAAATATCTTGTAAT<br>CAAGTTGATTCAGCAAAGTATATCTCCGTGATAAAGCATCAGCAATAA<br>TATTTTCCTTCCCTTTCTTGTGCTTAATAATATAAGGAAAAGATTCAAT<br>AAATTCAACCCATTTTGCATGCCTACGATTCAGTTTTCCTTGACTACG<br>AATATGTTTCAAAGATTCATGATCAGAATGGATAATAAACTCTTTGGG<br>CCACAAATAATGCTGCCATGTCTCTAATGTTCTAACAAGAGCATAGAG<br>TTCTTTATCATAAGTTGAATAATTGAGAACAGGCCCACTCAATTTCTC<br>ACTAAAATATGCAATAGGTTTTCCCTCTTGTAATAAAACACCTCCCAA<br>ACCAATTCCACTAGCATCACATTCAAGCTCAAAAGTCTTATTAAAATC<br>AGGAAGTTGTAGGAGAGGTGCATGAGTTAACTTATCTTTCAACATGTT<br>GAATGAATTCTCTTGTGCTTTGCCCCAATCAAAAGGCACCCCCTTCTT<br>TGTAAGCTCATTCAATGGTGCAGCAATGGTGCTGAAATCCTTCACAA<br>AACGGCGATAGAATCCAGCAAGTCCTAGGAAACTCCGCACCTGGGT<br>GATAGTATTTGGGACAGGCCATCCCTGTATAGCTTCCACCTTGGCTT<br>GATCAACCTCAATTCCCTGTGGAGTCACAACATAACCAAGAAAAGAC<br>ACTCGATCGGTGCAAAAGGTGCACTTCTCAAGGTTACCAAATAAACG<br>TGCCTCGCGTAGTGCATTAAAAACAGCACGTAAATGATCAAGATGTT<br>CATCCAATGATTTGCTGTAAATCAATATGTCATCAAAATATACGACAA<br>CAAATTTCCCAATGAAAGCACGCAAAACCTCGTTCATTAATCTCATGA<br>AAGTACTAGGTGCATTAGTTAACCCAAAAGGCATGACTAACCACTCAT<br>ACAAACCGAACTTAGTTTTGAAAGCAGTTTTCCATTCATCTCCCAATT<br>TCATACGAATCTGGTGGTACCCACTACGTAAATCAACTTTTGAAAACA<br>CAACAGCACCACTCAGTTCATCTAGCATATCATCTAATCGTGGAATAG<br>GGTGTCGATATCGAATGGTGATATTATTAATAGCTCTACAATCAACAC<br>ACATACGCCATGTTCCATCTTTCTTAGGCACTAAAATTACTGGAACAG<br>CACAAGGACTAAGAGATTCTCGGACATAACCTTTGTCTAGTAGTTCTT<br>GCACTTGTCGCTGAATTTCCTTTGTTTCCTCCGGGTTTGTCCTGTATG<br>GTGCACGATTTGGCAAAACTGCTCCAGGAATAAGATCAATTTGGTGC<br>TCAATCCCGCGTAGTGGAGGCAGCCCCGCTGGTACCTCACTTGGAA<br>ACACATCAGAATACTCCTGCAAAACGTTAGCAACAGCAGGGGGCAAA<br>GAACATTGCATATCCTCAATTGAAATCAAAGCATCCTTGCATATCAAA<br>GCATAAGCAACAGAAGTGGATGCATTGAACTCATTAATATCTGATTTG<br>GTAGCTATCATACAACGTCCTTTCAGTTTTATCTCATCTTTGTTACCAA<br>TTACAGATTTATCATTTTTATTGCTCTCGCTCTTTGCTTTAGTAGCCCT<br>AGCAACATCAGTTTGCATAATAGTTTCAGGGGACATAGGATGCAACA

	CAATTTTGCGATCATGGTATAGAAAAGAATACTGATTTGATCTACCAT GATGCATAGAATCTCTATCAAATTGCCAAGGTCTACCTAGCAGAATGT TACAAGCTTGCATAGGCACAACATCACATTCAACAATATCTTTGTAGG ATCCGATGGAAAAATTAATTCTCACAAGTCTAGTTACCTTTGCCTTAC CACTGTTGTTCAGCCATTGGATGTAGTAGGGATGCGGGTGTGGTTTG GTGTTGAGGGCAAGCTTCTGCACCATATCGCTGCTTGCCAAGTTGTT GCAGCTACCTCCATCAATGATCATGCGACAAGAACGCTCTTTGATGA CACACTTTGTTTGGAATAAAATGTGTCGCTGATTTTGCTCCGCCATCT CCATTTGTGCACTAAGCACTCGCTGCACGATCAAGCTCTCATAGTGG TCCGCTTCACCTGCATTAATATGTTCTTCTATTTGATCTTCATTACCTG CATGGTCAGTCGCAAGCAGTGCAAGTGTATCCTCATCAAAATCACTA GCAGATGAGTACTCACCATCATCCCTGACAATCATAACTCGCTTGTTT GGACAGTCACGCATCATGTGCCCATATCCCTTGCACCGGTGACATTG TATGTTGCTTGTTCTACCCGTCGATGCCACGGATGAAGTACTCGCAG CAGGCTTTTGCATCGTCTTCGCAACTGAATTGGTGGGGGCAGCTCGA GTCTTGTCACTAGATGATGGAGTAGAAATACGTGTAGATGGAGTTGA AGCCATGCGTTGCTGCCATGAATTAGCCTTCCCTGCAGAAATATTAC TCCTTGCGCTAGCACGTCGTCCCTGCACTTCCCTTTCAGCTTTACAA GCAAGATGAAACAAACGGGTTACATTATTGTATTCTTTGTAAGCGAGG ATGTCCTGAATTTCCCGATTTAACCCACCCAAAAATCTAGCCATAGCC GGTTCCTCATCCTCCTCTAGGTTACAACGCAACATACCCGTTTGTAAT TCCTGATAATATTCTTCTACACTTTTAGCACCTTGTCTTAACTGCTGCA ACTTATTTATCATATCACGCGCATAGTAAGATGGAACAAATCTAGCTC TCATGGCCCTTTTCAGCGCATCCCAAGTTCTAGGTAAGTTATTAGGAT TTTTCTTTCCATATTCTATCCACCAAACAGAAGCAAAATCTGTAAACTC ACTAGTAGCAGCCCTAACACGTGTAGTCTCAGGAAATTCATGACATG CAAACTTTTGATCAACAGCAATCTCCCAAGTGATGTAAGCATCAGGG TCATATTTACCATCAAAAGGGGGTATCTTAAATTTAATCTTACTGAAAG CATCATCATTATTGTGTACCTCGCGTCGGCGGTTGCCACCCATACCT CTACGGTTGTGACGAAGGCGACGTCGATCACGAGTGTCTTGATCATC ATGTTCAGTATCACCAGTGTAGTCATCTTCATGACTACCGTGCTCTCG ATCTCCCTCCTTTTCTTCTTTATGCTTCTCTTTATCTTCGGTGTGGAAA TCATCAAATCGCCTTAGGAGAGCAGCAAGGCTTTTGTCCACACTAGC AACGGATTCCTCCAAACTTGTGAGCTTGTTGTTTGTGGCAATCTGCG TAGCCTCCAACTGCCCCAGCTTTTCATTTGTCACCTGCAAGTCGTTAT CAAGTCCCTCTGTGTGCAGCTTCACTTTCCTTTCAAAATGTTGTATGA TGCCCTTAGTGCGAGGTGTAAGTGGTGTTTCGTTACCATCATCTGCC CCTGGCATGGTTCAAAGACAAAGACAACAAAAAGGCAAGTGAAGAAA TAAAAGCCCTACAACTACTAGGATGTAGCTACAGCAAGTCGCTCACA CTCAACCTGTAACACAAGTTCTTACCAATTCTTACCTTGCTTGACAGG AGGGGTCGTCTGCCAACAAGTGTACAGCAATGGACGAAGTGTATCG GTGCTGCAGCACAAGACCTGTCAAGCTGTAGAGTATGTGGAGCTATA GGTGGGCTGAAACAAGGAACGAACTAGCACCACGTTAGTTATAAAAG CGAGCTGAATAAGCGTTCGACGATGGTACTGTGCTGGTCCTAGGCTA GACCGTGCTAGAGACGCGAGCCTGGACACAAAGGAAAACCACAGCA CACCCCCGAAAACAAGGTGGAACAAACCTAAAATCAATATGAAAAAC AGCCCCTTTTTTTTAATTTTTTCTCTTTTCTTTTTTTTTTTTTTTTTTTCT TTTTTTTGGGCAACCTCAAAAACTGATTATATGAACAAAAGAATACAA AAGAGCAATTCGCTACACACACTTTTTTTCTCTCTCTCTCTAGAGTAA GGCCGAACAAGTA
SorCRM_LTR3 (CL143)	>SorCRM_LTR3_CL143 AATATATATGTGGAATCACTCCCTCACCACTTACAAAACAAGTTCTTT CTCTCCCTGAAAAGAGCAAGAACCGGGGCTGCGATCTGTAGGGAAG AGTGATTGTAACAGCGACGACGGTGCAACAACTCACGGTGCTTTAAG TGGAGCTTGGTGGGGTAATATGTTAGTGGAATGCACTGAAATGTAGT GATGCAAAACCAAATAATAATCCAAGAACAGAAGTCTAAGTTGCTGAA

	TATAAAGGAAAGGATGCACAAAGAAAGGAATGAGATGTAGTAAGTGG<br>ATAGATGATCACCAATTTGCACCAACCGAAAACTTGTTGCTGCCCAAA<br>CCCCTTTTTGGTGTGCTGCACACAGTTCTCTTTTTGTTTTTCTTTTTTT<br>TTCTCTTTTTTTTTGGAACAAAAACTCGTTTTCAGTCCTTCTTTTGACC<br>CATAGACATCTTTTCTTTTAACCTCTGTGTCAAACGCAGCACAACTTT<br>CAACTAAAGGGGATCACTAAGATGGATGGTGCAGCACAAAAACAAGA<br>AACTGCGGTGGGGAATATGTGGCGGTTAGAAAACAAGGGTGGGATG<br>GATGGGTACATGCAGCAAGCAATTTGATGATTAGAACAAAGATAATAA<br>CTAGAACAATGTGGAAAAGAAAAATTCAGCAACTAGACAAATATTAAA<br>GGATTAAAACTCGATAAAGCAAACTACAAAATCAGTAGCACATATGAA<br>TTTGGGCTGTAGGTTTTCTTTTTGGCTCTCAGTGGACAGTAGGTATTT<br>AAACTCTATCCTACCAAAATCAAGAACAATCCTACCGAGGAAACGAA<br>GGCTTTGGTACCAGATGATATGCTCACGCCTAGGGATAGCGTGAGA<br>CAAGTCGTTGTGGCCCGATCTTCTCGTAGGATTCGCGAACTTGATAA<br>CTTGTCGCTGCGTGACCTGGTGAAGAGGCAGTGCACCGCGAGGCTG<br>TCCACGCGACGAACTACCGAGACAATCACCCTTCCACGTACCGACG<br>AACAGCCCACGCAATCACGCCGTATAGACGTGAAGGCACAAACTGG<br>ATGAACCGCAGTTCGGCGTTCTACAACTCCCTCAGGAATCGAAAGAA<br>CAAGTTTTGCAAGCCTCTCAAGACTCACGCATAAACAAAACTCCACG<br>AGTTTGTGTATTCTGAATTTTAACCAAGCAAGATGTGTCCTTTCATTGT<br>CTAGTACAAGAGATATATATAGATAGGGGCGTTCAGCTTTGAATACAT<br>GACAGCATGCACATCCACTAGTGGATTTCGTGGCTGGTTCGCGCGT<br>CTTCTCAGCTTCTGGCAGCAGTTACTAAAATGAGCATAACTCTTTATT<br>GGGAAGTCTAAATAATGAACCGTTTGATGGGCTGCAACATAGACTTA<br>AAGATGCTTTCATCCATCTGTAGAATGCCACGTAACTCCTTGTATTCT<br>GTCCGTGGTGATGCTTGGAATTTGTACCATGGGTCAGCCATCTAGCT<br>TCTGGTTGCCTTCTCATGCAGAAGTAATGAACCACCATTTTATTTATG<br>CACACGATAGGCTTCTTGGTTCAGATGTCCAAAGGCTTGAATCCATC<br>TTCATCCCATGCTGGTTCATACCCTCCATCATTCCTAAGGACATTGAA<br>ACAAGAACTCAAAAGTATAGGATCATTCAACATAAACTGATTATTAAA<br>CATAAGGTTAGCGTTCACCTGAGAATGAATTTCCTTCTCCAATTGTTT<br>AGCTCTACTTCTTGTAATTGGACCACTTATATCAAGTGGAGTTTCATT<br>TGAAGATGAATGAATGCTAGGGATGTCCTCATCAGCCTCCCCCTCTT<br>GAGAAGGAGTCGTCCTCGACTCAGGCTCACCAACAAATGGAAGCAA<br>GTCTTTGACATTGAATGTTGGACTAACATTGGAATATTCAGGTGGCAG<br>CTCAATTTTGTATGCATTATCATTTATCTTTTTTAGAACCTTGAATGGA<br>CCATCACCTCTAGGTGATAACTTACTCTTACGTTGTTGGGGAAATCGA<br>TCCTTGCGTAGATGCAACCACACGAGGTCACCGGGCTGAAATGTAAC<br>CTTCTTCTTACCTTTGTTCGCTTGCTTTGCATATTGTGCTGATTTCTTC<br>TCAATATTTCTTCTTGTCTCATCATGTAGCTTCTTGATAAAGTTGGATC<br>TCTTCGCTGCATCCAAGTTAACTTGTTCCTGTAATGGTAAAGGCAAAA<br>GATCCATGGGAGTATGTGGCTTAAAACCATAAACAATTTCAAATGGAC<br>AAAAATTTGTTGTGGAATGTAGTGCCCTATTATAAGCAAATTCGACAT<br>GTGGAAGACATTCTTCCCACTGCTTCAAATTCTTTTTTAGCACGGCAC<br>GCAGCATGGTGGACAGGGTACGGTTGACAACTTCAGTTTGCCCATC<br>CG**TTTGTGGGTGACAAGTGGTG**GAAAATAACAACTTCGTGCCAAGTT<br>TAGCCCATAATGTCTTCCAAAAATAGCTCAGGAATTTTGTATCTCGGT<br>CTGAAACAATAGTCTTTGGCACTCCATGTAAACGAACAATCTCCCTGA<br>AAAACAAATCAGCAACGTGTGAAGCATCATCGCTCTTGTGACATGGA<br>ATAAAATGTGCCATTTTTGAGAAGCGATCAACAATAA**CGAAGATAGA**<br>**GTCCCTCCCC**CTCTGAGACCTTGGTAATCCCAGAATAAAATCCATGG<br>ATATATCTTCCCAAGGTACAGTTGGGATTGGTAATGGAGTATAGAGA<br>CCATGGGGATTAAGGCGGGACTTAGCTTTCAAGCAGATTATGCAGCG<br>CTCGACATGTCGTTGAACATCACGTCGCATATGTGGCCAAAAGAAAT<br>GATCAGAGAGCATGTCCAATGTCTTTTTGATGCCAAAATGACCAGCT<br>AAGCCACCAGCATGTGCTTCCTGTAAAAGAACTTGACGAATCGAGCA

	GGCTGGAATGCATAGTTTGTTAGTTCGAAATAAAAAATCATCATGTAC<br>ATAATACTTGTCCCAGCCTTTTCCAGCAATGCAATGAGAGAAAGGTTC<br>TTTAAAATCAGAATCAGTTGCATAGAGTGTTTTAATGGATTCCAAACC<br>AAGCACTTTTGTATCAAGTTGTGTAAGCAAAGCACAACTTCGTGACAA<br>AGCATCAGCAACAATGTTATCTTTACCATGCTTATATTTAACAACATAA<br>GGAAATGATTCAATGAACTCACTCCATTTTGCATGTCTTCGATTCAGT<br>TTGCCTTGACCTTTAAAATGTTTCAAAGATTCATGGTCAGAATGGATG<br>ACAAATTCTTTAGGCAAAAGATAGTGTTGCCAAGTTTCCAAACTTCGA<br>ACTAAGGCATAAAGCTCTTTATCATAGACAGAATAATTCAGAGGTGGA<br>CCATTTAACTTTTCAGAAAAGTAGGCAATAGGTTTACCTTCTTGAAGT<br>AGCACACCTCCAATGCCAATACCACTTGCATCACATTCAATCTCAAAA<br>GTCTTACCGAAATTAGGTAGCTGCAGCAGTGGTGCTTCACAAAGCTT<br>TCTTTTTAACTCTACAAAGGCTTGCTCTTGGTCATCTCCCCATTTGAA<br>TGGAACATCCTTCTTTGTCAAGTTGTTAAGTGGTGCAGCGATCGTGC<br>TGAAATCTTTAACAAATCGTCGATAGAAACCTGCAAGACCATGGAAAC<br>TTCGAATTTGGCTCACATTTGTAGGAGTAGGCCAATCCTTTATTGCCT<br>TAACCTTCTCTTCATCAACCTGGATGCCATCTGCAGTCACAACAAAAC<br>CAAGAAAAACAACACGATCTGTGCAAAATGTGCACTTAGCAATGTTG<br>GCATACAATTTCTCTTCCCTCAAAACAGCAAGTACTTGATGGATATGA<br>TCAAGATGTTCATCAAATGATTTGCTATAGATCAGAATATCATCAAAAT<br>AAACAACTACAAACTTGCCAATGAAAGCTCGTAAAACATGATTCATTA<br>AGCGCATAAAAGTTGAAGGAGCATTTGTCAAGCCAAAGGGCATCACA<br>AGCCATTCATACAAGCCAAATTTGGTTGTAAATGCTGTCTTCCATTCA<br>TCACCAATTTTCATGCGAATTTGGTGATAGCCACTGCGTAAATCAATC<br>TTGGTGAAAATAGTTGAGCCGCTCAATTCATCTAACATGTCATCAAGC<br>CTAGGAATGGGATGGCGATATCGTACAGTTATAGCATTAATGGCACG<br>ACAATCGACACACATGCGCCAAGATCCATCTTTCTTAGGGACCAAAA<br>GTACTGGAACAGCACAAGGTGATAGAGATTCACGAACATACCCTTTG<br>TCCAAAAGCTCTTTTACCTGTCGCTGAATTTCTTTGGTTTCTTCAGGA<br>TTAGCACGGTAGGCTGGACGATTGGGAAGAGAAGCTCCAGGTACCA<br>AATCGATTTGATGCTCAATACCACGGAGTGGAGGAAGGCCAGCTGG<br>TACCTCATCAGGAAAAACATCTTCAAAGTCCTGTAAGAGATCAAGAAC<br>AGCACTAGGCAGCGATGAAGGTAAATCGTTAGTTGAAAGTAGGACCT<br>CCTTGTGCAAGAGCACAAAGAATGGGGCTGTGGTGTTCCTCACTTCT<br>CTCAAATCACTCCTGCTCACAAATAAGCACTCATTTTGGTGGTGTTGT<br>TTTGTAGTGGAAAGAGGCTTTATGTGGCTAGATTGGTTAGAAGTCTCT<br>CCCTTACTAGTGTTGGCAGCCTCACTCAATTTTCTTTTGTCAGATTCT<br>TCTTTTTTCATGCGAGCCACATCTGAAGCATAGATCTCTTCTGGAGAT<br>AATGGAACAAGAACCACCTTCTTGTCGTTGTGGATGAAAGTATACTTG<br>TTAGACCGCCCAAAGTGCACCGAATCCACATCAAACTGCCATGGACG<br>ACCCAACAGCAAATGGCATGCTTGCATGGGTACAATATCACAATCAA<br>CCTCACCATGATAGTCACCAATGGAGAAAGACAAACGAACCATGGAT<br>GAGACCTTCACTGTCCCTGAATTATTCAGCCACTGCATATGGTAAGG<br>GTGTGGATGGCGGCGTGTTGGTAGGCCAAGCTTCTCAACAAGCAAG<br>GCACTAACAATATTATTGCAGCTCCCACCATCTATGATGAAACGACAC<br>ACTTGACCTTTCACTTTGCATCGGGACTGAAACAAATTATGACGTTGT<br>CCTTGTTCAGCAGCAACAAACTGAGTGGAAAGAACTCTTCTAACCAC<br>CAAATAAGGAGATGATCTATTCATTTTAGAAGGCTCCAAATCAGGAAA<br>ATCAGCAAGCAACTCATCAAAATCAGCACTAGTAATCTCATTAGAATA<br>TGGAGAAATAACATCTTCTATCATGTCACTGGGAGCAAAAGTTAGAAT<br>AGGTTGAATAGCTAAACAATTCAGACCTAGCTCAAAAGTACCATCCTC<br>AGCTGAATACTCACAAGTGTCAAGAGTATGATCTGCAAAGACATTGT<br>GAAACTCGTCCTCCTCTTCACTTTGTGAATCATATGAACCATCAGCAA<br>GGGCAATAATCGTACGACGATTGGGACATTCAGCTTGCTTATGCCCA<br>TGACCACCACACTTAAAACACTCAATCTTGCTTGTATGCCTTGGGGCT<br>GCAGTAGCAGAGGAGCTGGACTGAGTAGAGCTTACAGCTTTGCCTTT

	GGAATCAAAATGTTTGGAAGAAGTTGCACGAGATGTAGGTGTGTGCA<br>CCCCTGACCCGTGCTGCTGTGACTGGCGCCATGAAGTAGCACTATTA<br>TGAGCTGAAAATGAAGCACGATCTTTATAAGATCCAGCAAGTTGTCG<br>TTCTGCCCTTTTTGCAAAATGTACCAACTCAGTGAGACATGTGTAGTT<br>TGTCATATCCACTTTATCAGCAATGGGCTTATTGAGGCCAACCAGAAA<br>TCGAGCCATTGTGGATTCCTCATCTTCAGTTATCCCTGTACGAAGTAA<br>ACACATTTCCAATTCTTGAAAATATTCATCAACAGTACGAGTACCTTG<br>CACAAGACGTTTTAGCTTCAAATGTAGATCACGAGAGTAATATGCAG<br>GAACAAAACGACGTCGCATTTCCCTCTTCATGTCTTCCCAAGTAATAC<br>GATCATGTCCAGCGCGTCGGAGTTCAGTACACACTTGATTCCACCAA<br>GTGATTGCATAGCCTGAAAACTCAATTGCAGCAAGCTTTGCTTTCTTG<br>ACAGGAGGATAAGGATACAAATCAAAGATCTGTTCAACCTTAGTCTC<br>CCACTCAAAATAATCATCAGCACTTTCTTTTCCATTAAATTTTGGAATA<br>GACACTTTCACTTTGCTTAAACCATCATCATCAAGGCGACGGTGGCG<br>GTCACGATAACCACGATGGCCACCATGGCGATGATCATCCATGTCAG<br>ACAACATATCATCATCACCATCATAATCTCGTCCACCTCGAACAGGAA<br>TGCGCCGAGCACGACCAAAATTAAGAAGACCATGACCACGCCCGCC<br>ACGTCCACGACCAGCGGGACGACGCGGTGGCTCATCCTCCTCGTCA<br>ATATCATCATCCTCATTTGGTGGCGGTTCTCGACGTGGTATGTTCAAT<br>TGGAGAGTTTGGAGCTGCTGCACCAAATCATTCATTTGTGTACGCAA<br>CTCCTGAAATTCCTCCACCGAAACAGCGGCACCATCTCCACGTCCTG<br>CCATGTTAGTAGAGGAAAAAAAAGGAC
SorCRM_LTR4<br>(CL164)	>SorCRM_LTR4_CL164<br>GATGTGGTTGTTGTAATTATTTCACTCTCACCCTATGCAAACAAGTTC<br>TCACAAGTTCTTACCAAGCGAAACAGGAGGTGGCAGGAACCAACAA<br>GTCAGAAAACCTAGAAAATGGTGTATCAACGCTGCAGCAACAACACT<br>TGTCAAGTTGTAGTCTGATATGTGGAGTTATAGGATGGCTCTATATAT<br>TGCACAAGGAAGTACTAGCACCACATTAGTCACAAAAGCATGTTGAG<br>AAATCATTCAACGGTGGCACTGTGCTGGTCCTAGGCTAGACCGTGCT<br>AGAGACGCGAGCCTAGACACAAACAAAGTCACGGTACAGCAGGAAC<br>AATAATAAGGACAACAAGAGATAACTTCCCTTCTTTTCCTCTTCCTTTC<br>TTTTCTTTCCTTTTTCTTTTCTTTTTTTCTGATATTTTTTTGTTTCTTTCT<br>GTTTTTTTCCTACTTTGCTCAACTAAAATAAATCTGAATCTTGATGGAT<br>TAGATTTCAGAAAACACACAATGTCTTTAGTGTAACAAAAATACAACT<br>AGCAGATTATGATGGTGATATGATGCTGCAGTAACACTGATTGCAAC<br>GGGGATGAGAAAAATAGAATGTGAAAGAAGAGTGGAGGAGAGGCAA<br>AAGGATGCGCATGAAGTGGACAAGGATGCAATGTTCTAAAATTAGAT<br>GCCCAAGAGTGGAATGAATCTAGTGGTTGCAATAGGATGCTGATTAT<br>AGAATGTGCAAGAGCGCTTACAATAGGATGTATGAATGGAAGAAAGG<br>AACATGCAGGTGGCAGTGAGTAAGAATAGGTTGCTCACGGGTGGAA<br>GTGAAAACGTGCATGCAATGGCCTAAAAATATAGGGTGCGCATGAGT<br>GAAAAGAGAGTGGTGGATGGAGTTTACACGGTGCCCAAGTAGGGTG<br>GTGATGGTAGTTCAGATATATAGTGTAGTCCAAATGTATATGGTAATA<br>GTGATTGCAGCGGAGATTATACGATGGGCAATGACTAAAACGTGACA<br>AAAACTCGAAACTCTAAAAGTCTAAACCCAAGACCAGCAATTCGACTC<br>GACCGATGTAAATCAAACACAACTTGCCTGAACTAAGTAGTACTAGTA<br>AAAGGCTCAATGGGTCTATAGGATGTTGGGAATGAAACTAAATTTTTT<br>TTGGCTTTTCTGGACTATAGGAAAAAAAATTGCAAACTAAAATCTCTC<br>ACCAATCAAACCTTGCTCTGATTACCACATAATAAGAACGAGGGTGT<br>CCCGATCTTTCGGTGGAGATGGATAACTTCGATCTCTCTGGAGCGAC<br>GTTCACGATCCGACTACAACCGTCCAAGGACGCTGCGCCTTAGCGA<br>TCGATACACCAATTCCAGCGGTTGATCACGATCTTGTTGATCACAAC<br>CAACCAAACCACTAGGGTAATTCCTGCACGCAATCGAAGAACATGCA<br>AGAACAGATAGAACAGGCACTCAATTGCCAAATAATACAAGAAGGAT<br>CTGATATGAATCACGATAACAATATGGGGTTTTGAATCAAGTAGAACG<br>GATGGTCTAGTCGACACATGCGTCTACAAGGAAGTAGCAATGGCTAA

	ACTTACAACAAAACAAAACCCGCCTTGTGTCTCTGGCTGTAGAAGCC<br>TTTATGTACAAGAGAGAGCAACCAGAGTGTGCGGTGCTAATCCCTCG<br>ACTCACTAAAAACAAAAGAAAACAATAATATCCATATCATGCATGCAT<br>GGAGTTTCTTATTGAATGAAGCTGTCCACAGAAAGGTCTCTGGAGTA<br>ACTCGACGATTGCGGCCGCCTCATTATAGATATGGACATGGGACTGG<br>ATCCATGTGAAAATAGACTTCATAAGCTTTCCACCGTGTATTTGAACA<br>TCAAAATCGGTCACCGTATGTAGTCTTGGTGGCCACCACAAGTTGAG<br>ACCCTTCTGCAGTCCAAATTTAGCCCATCGCGAATATGCTTCCTTGC<br>GGTTCCTCCTCATTATTCTAGATAGTTGAGTTATCATGCATATGAATC<br>TGAGGCGTGGATGTATTGATGGTGCTCATGTCCTCATCATCCTCCCC<br>TTCTTGCATTTGAGTCGTCCTCGACTCAAGCTTGTCCTCTTCTCCCAA<br>GTAGGGCATAATGGTCCCCAATCAACTTCATTCACCATCCATACTTTC<br>AATATGCAATCAAATATTCTCCTGATCCATGTAAGTATTACGGAGCAG<br>GTGATGTCCTCATCATTCTCCCCTTCTTGACAGCAAACCGTCCTCGG<br>TTTAATCATTGGTGGAGCATGTGTTGTCAGAATTGATGAACCTTGCTC<br>TCCTGCTTGATACTTGCCCTGTTCCATCATAAGAAAACTAGGCAAACC<br>CGCCATGACATGGTTATGGTTATTGCACACTTCTAGTTGGTCATATTG<br>TTTCACAATAAAAGGAGATTTTATGTTTGAATAAATGTAAATTCGGTGT<br>ACCATATACTCCTTTGCATAATTATATTTGCCAATTGCATGAAAACTAA<br>CATTGGAAGAGCATGACAATTCAAATCTAGAAAACTGATTATTCTCTA<br>AATAGCTAAGCTCACAAAGATCATCGAATTCAATGTACCCCATTATAT<br>GTAAAGATGACAACAATTGAGCTTCTTCATTTTCATTTACAATATTAAT<br>AACATGCTTGAATTCGACACTCATTGATTCAGAATTCTTTTCTTTGTAT<br>ACTAGTTGTGGCATGGATATAGGTGATGCACTATTGCACAACTCTTCT<br>TTGTCACTAGGAACATCAGGAATATCGAAGTTTGACAAAGGAAAATTA<br>GTAATTGGTTCCATAATTGTTTTCTCTAAGTTAGCAGCATTAGTGGAC<br>AAATGCAGCTCATCTAATGCAAGGTCACCTTGATTTATGTTACCACCA<br>TCTAATTTACCTTTGATGCTGTGTTGAAATGGTGTGGGTGCAGCTAGA<br>TTTACGCTCTCTTTAAATGACTCATCTAGTATCTCAAGTTGCACGCTTT<br>CCATGTGTATGTTGTCTTCTTGTTGCTGCAAATTGTTAGTAATAGGAG<br>AGACACGTACTTCTATAATACTTGCGAGATCCCTTTCTAACTCACAAG<br>CAAGATCAAACAATTCATCAAAGGTGTTATAATACTTATCAACAAGAA<br>TGTTCTGAATTTCTACATTAAGACCTTGCAGAAATCTATGTTCCAAAA<br>GTTCATCAGTTTCT**TCTAATCCACATCGCAGCAC**ACACAATTTTAGTT<br>TATCCAAATATTCCGCTACGGTTTTGTTTTCTTGCTTTATAGATTGTAG<br>TTTTTCAAACAAAATTTCAGAATAGTACTCAGGGACAAAAACATCTCT<br>AAAATGTCTTT**TCACATCTTTCCATGTTTGAGG**AGCTTTATGAAATCT<br>ACAAAGGTATTTCCATGAACTTAAAGCATACTCAGTAAGAACACTGGA<br>AGCAACTCTAATCTTTTGGCTTTCAGATAGATTATGCTTTTTAAATTCA<br>TTGTCTACATTTAGCTCCCATTTAATATATGCATGTGGATCACAATTTC<br>CTTCAAAATAAGGTAATGCAGATTTTATTTCATCTAGCAAAACATCACA<br>TACCTCATGAGTGCCATACTTATCACTTGCCACACCTTTAGCAAAGTG<br>ATGTGAACATGCAATCTGATTATCGCTTGTTTCTACGTGGATTCCTGC<br>CATGATTAGAACAAAACAAGAGACATACGCAATAATCTTTATGCTCCT<br>ATCAACTACAAG

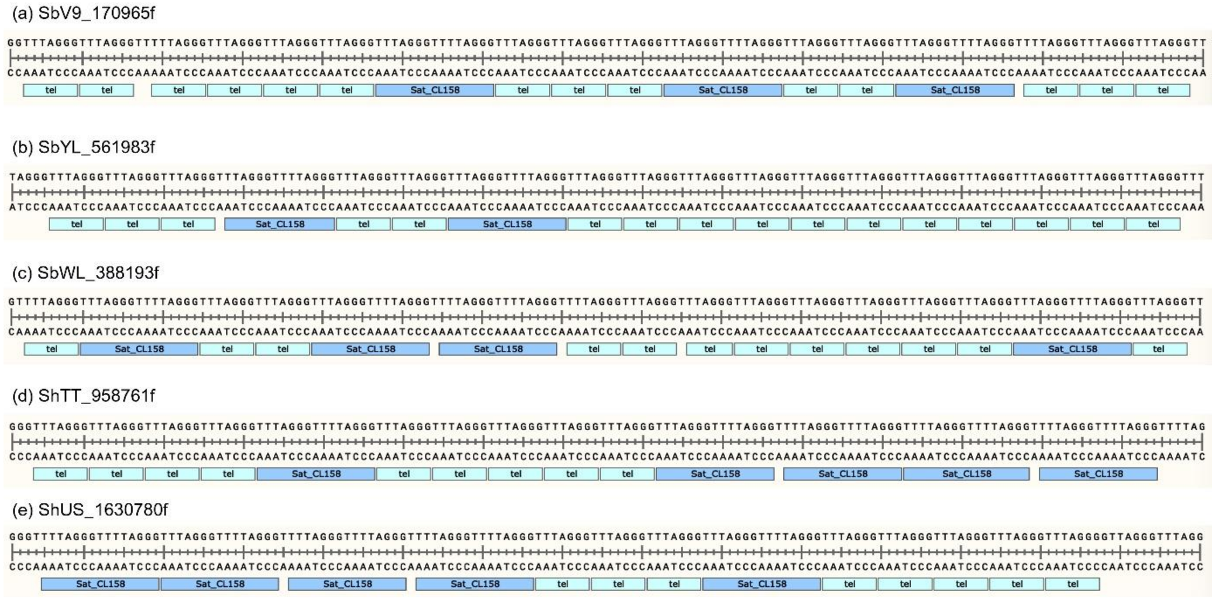

Supplementary Figure 1 Distribution of the satellite repeat CL158 and the *Arabidopsis*-type telomere. One satellite repeat CL158-containing Illumina read from each *Sorghum* accession was selected to analyze the sequence composition. In all five *Sorghum* accessions, the satellite repeat CL158 (Sat_CL158, blue box) either intermingles with or tandemly arrays next to the canonical plant telomeric repeat (tel, green box).

```
SorSat200_ShTT      TCTAGACTTTTCCATACTTTTATGTGTCCAAACCATAGTAAAATTCCATGTGTTCAAGGT     60
SorSat200_ShUS      TCTAGACTTTTCCATACTTTTATGTGTCCAAACCATAGTAAAATTCCATGTGTTCAAGGT     60
XSR3_X54624         TCTAGACTTTTCCATACTTTTATGTCTCCAAACCATAGTAAAATTCCATGTGTTCAAGGT     60
XSR1_X54623         TCTAGACT-TTTCATCCTTTACGTGTCCAAACCATAGTAAAATTCTATGTGTTCAAGGT     59
XSR6_X54625         TCTAGACTTTTCCATAATTTTATGTGTCCAAACCATAGTAAAGTTCCATGTGTTCAAGGT     60
                    ******** **       **** ** **************** *** *************

SorSat200_ShTT      ATGGTAATATTTTTT-TGTGAAAGCTTGAGGACCAATAGTTCATAGCTAGAGGGACCCCA     119
SorSat200_ShUS      ATGGTAATATTTTTT-TGTGAAAGCTTGAGGACCAATAGTTCATAGCTAGAGGGACCCCA     119
XSR3_X54624         ATGGTAATATTTTTTTTGTGAAAGCTTCAGGACCAATAGTTCACAGCTACACCCA-CCCA     119
XSR1_X54623         ATGATA-TATTTTTTTTATGAAAGCTCGAGGACCAATAGGTCATAGCAGAGGGAC-CCTA     117
XSR6_X54625         ATGGTAATATTTTTTTTGTGAAAGCTTGAGGACCAATAGGTCATAGCAGAGGGAC-CCTA     119
                    *** ** ******** * ********  *********** *** ***         ** *

SorSat200_ShTT      TCACACCTCGAGGGACCCTGTCACAGCTCGAGATAGCCTATCACAGCTGAAAAAGTCTAG     179
SorSat200_ShUS      TCACACCTCGAGGGACCCTGTCACAGCTCGAGATAGCCTATCACAGCTGAAAAAGTCTAG     179
XSR3_X54624         TCACACCTCGAGGGACCCGTCA-CAGCTCGAGATAGC-CATCACAGCTGAAAAGTATAGG     177
XSR1_X54623         TCACAGCTCGAGGAGCCATCAT-A---------------------GCTGAAAAGTCTAGG     155
XSR6_X54625         TCACAGCTCGAGGAGCCATCAC-A---------------------GCTGAAAAGTCTTGG     157
                    ***** *******  **                            ********      *

SorSat200_ShTT      GACTCCAAACCATGGTAAAAT------ 200
SorSat200_ShUS      GACTCCAAACCATGGTAAAAT------ 200
XSR3_X54624         ACTCCAAACCA-TCCTAAAATTCTAGA 203
XSR1_X54623         ACTCCAAACCA-TGGTAAAATTCTAGA 181
XSR6_X54625         ACCTCCAAACTATGGTAAAATTCTAGA 184
                        * ** *  *  ******
```

Supplementary Figure 2 Sequence alignment of the satellite SorSat200 and the reported *S. halepense*-specific XSR repeats. The consensus sequences of SorSat200 from two *S. halepense* genotypes, ShTT and ShUS, are identical. The XSR repeats XSR1_X54623, XSR3_X54624 and XSR6_X54625 (EMBL-EBI (https://www.ebi.ac.uk/)) were identified by (Hoangtang et al., 1991). Sequence alignment was performed using Clustal Omega (https://www.ebi.ac.uk/Tools/msa/clustalo/) implemented in EMBL-EBI.

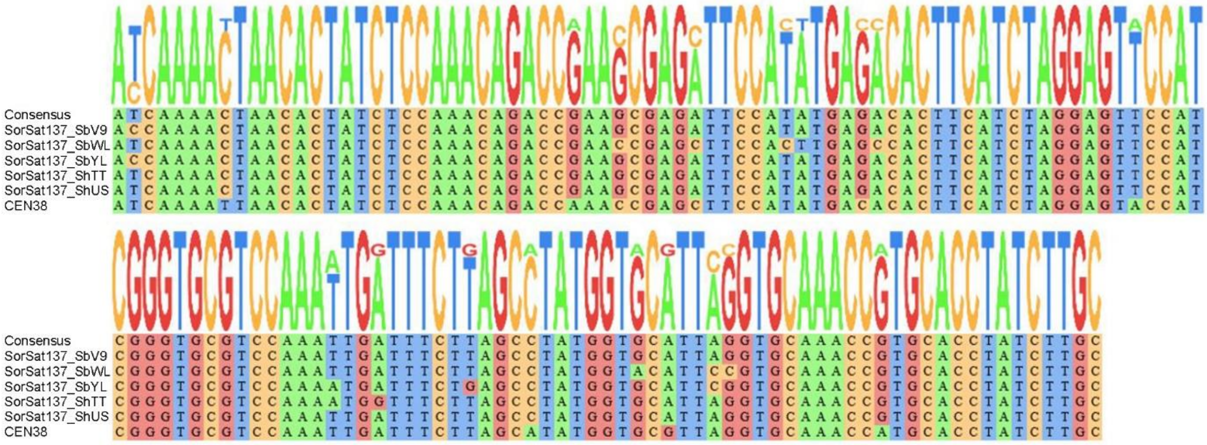

Supplementary Figure 3 The centromeric satellite family SorSat137 is conserved in *Eusorghum*. Sequence alignment of the SorSat137 family members encoded by *S. bicolor* (SbV9), S. *bicolor* ssp. *verticilliflorum* (SbWL), *S. bicolor* var. *technicum* (SbYL) and S*. halepense* (ShTT and ShUS). The consensus sequence of the SorSat137 family, which was deduced using the software MAFFT (Katoh and Standley, 2013), and shown above the alignment. SorSat137 represents the centromeric repeat CEN38 of *S. bicolor* (Miller et al., 1998a; Zwick et al., 2000)
